# Xenogeneic skin transplantation promotes angiogenesis and tissue regeneration through activated Trem2^+^ macrophages

**DOI:** 10.1126/sciadv.abi4528

**Published:** 2021-12-01

**Authors:** Dominic Henn, Kellen Chen, Tobias Fehlmann, Artem A. Trotsyuk, Dharshan Sivaraj, Zeshaan N. Maan, Clark A. Bonham, Janos A. Barrera, Chyna J. Mays, Autumn H. Greco, Sylvia E. Moortgat Illouz, John Qian Lin, Sydney R. Steele, Deshka S. Foster, Jagannath Padmanabhan, Arash Momeni, Dung Nguyen, Derrick C. Wan, Ulrich Kneser, Michael Januszyk, Andreas Keller, Michael T. Longaker, Geoffrey C. Gurtner

**Affiliations:** 1 Hagey Laboratory for Pediatric Regenerative Medicine, Division of Plastic and Reconstructive Surgery, Department of Surgery, Stanford University, Stanford, CA, USA.; 2 Department of Hand, Plastic, and Reconstructive Surgery, BG Trauma Center Ludwigshafen, Ruprecht-Karls-University of Heidelberg, Heidelberg, Germany.; 3 Chair for Clinical Bioinformatics, Saarland University, Saarbrücken, Germany.; 4 Department of Neurology and Neurological Sciences, Stanford University, Stanford, CA, USA.

## Abstract

Skin allo- and xenotransplantation are the standard treatment for major burns when donor sites for autografts are not available. The relationship between the immune response to foreign grafts and their impact on wound healing has not been fully elucidated. Here, we investigated changes in collagen architecture after xenogeneic implantation of human biologic scaffolds. We show that collagen deposition in response to the implantation of human split-thickness skin grafts (hSTSGs) containing live cells recapitulates normal skin architecture, whereas human acellular dermal matrix (ADM) grafts led to a fibrotic collagen deposition. We show that macrophage differentiation in response to hSTSG implantation is driven toward regenerative Trem2^+^ subpopulations and found that hydrogel delivery of these cells significantly accelerated wound closure. Our study identifies the preclinical therapeutic potential of Trem2^+^ macrophages to mitigate fibrosis and promote wound healing, providing a novel effective strategy to develop advanced cell therapies for complex wounds.

## Teaser

Vitamin D–activated Trem2^+^ macrophages provide a novel strategy to develop advanced cell therapies for complex wounds.

## INTRODUCTION

For more than 50 years, transplantation of unmatched human skin grafts has been the clinical standard of care for major burn wounds when autologous skin grafts are not available because of the extent of the burn injury. Although the cellular elements of unmatched grafts are rejected by the host (*1*), extracellular matrix (ECM) elements integrate into the wound bed and are potentially beneficial to wound healing in patients with burns (*2*–*4*) and chronic wounds (*5*, *6*). In mice, it has been shown that the implantation of xenogeneic dermal scaffolds does not induce a strong inflammatory response but has a strong impact on macrophage polarization, potentially inducing regenerative phenotypes (*7*, *8*).

Macrophages are a heterogeneous and highly plastic cell population and believed to be critical mediators of the cellular response during all stages of soft tissue injury. Traditional classification systems of macrophages describe “classically activated” M1 macrophages, dominating the acute inflammation stage after injury, and “alternatively activated” M2 macrophages, which are thought to be involved in reparative processes of soft tissue remodeling. These classifications, however, were derived from in vitro studies and have been shown to insufficiently characterize the broad spectrum of macrophage phenotypes found in physiologic and pathologic conditions in vivo (*9*, *10*, *11*). Implantation of both synthetic and biological materials induces specific tissue microenvironments that likely drive macrophage subpopulations. Synthetic materials commonly instigate a proinflammatory response, whereas biological grafts can induce regenerative macrophage subpopulations (*7*), potentially suppressing the development of fibrosis by producing arginase 1 (ARG1), resistin-like molecule-α (RELMα), and interleukin-10 (IL-10) (*9*, *12*, *13*). To add to the complexity, other studies have suggested a profibrotic role for “M2” macrophages because of their ability to stimulate fibroblast differentiation into myofibroblasts (*9*, *14*, *15*). Collectively, these findings indicate that the impact of macrophage heterogeneity on the process of wound repair and tissue fibrosis remains incompletely understood.

Here, we examine the impact of clinically used human biologic scaffolds on the innate immune response and wound healing over time in a murine model. Using single-cell RNA sequencing (scRNA-seq), we show that macrophage polarization in response to cellular human split-thickness skin grafts (hSTSGs) is driven toward a distinct subpopulation characterized by a high expression of the lipid receptor *Trem2* (triggering receptor expressed on myeloid cells) as well as proangiogenic and antifibrotic gene expression profiles. We then confirmed that vitamin D (VD) signaling drives clonal proliferation of myeloid cells during skin healing using myeloid cell–specific Cre-mediated recombination in a multicolor reporter mouse model. Last, we developed an approach to induce regenerative Trem2^+^ transcriptomic programs in vitro by treating bone marrow (BM)–derived macrophages with vitamin D3 (VD3; 1,25-dihydroxycholecalciferol) and showed that Trem2^+^ macrophages significantly accelerate the healing of murine full-thickness excisional wounds.

## RESULTS

### Cellular human skin grafts remodel into a physiologic dermal collagen network after long-term implantation and attract regenerative macrophages

**Fig. 1.**
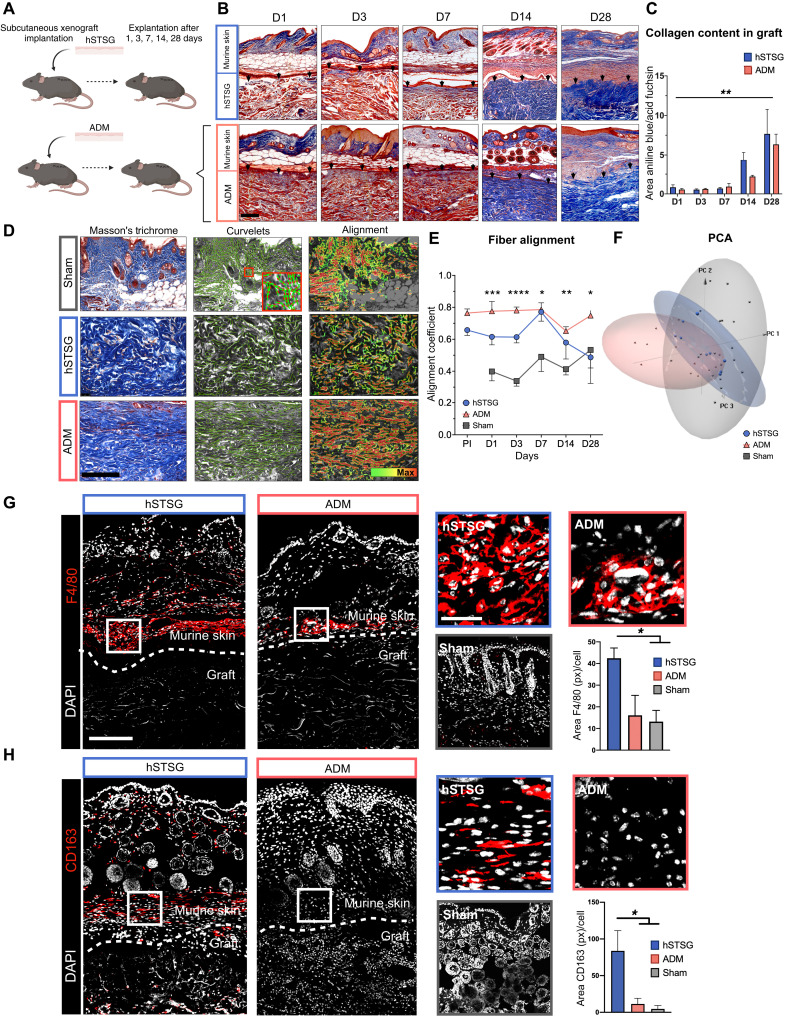
hSTSGs remodel into a physiologic dermal collagen network after in vivo implantation and attract regenerative macrophages. (**A**) Xenograft implantation model (*n* = 6 to 8 per group). (**B** and **C**) Masson’s trichrome staining of explanted grafts with overlying murine skin. Black arrows indicate the interface between graft and skin. (**D**) Left: Masson’s trichrome staining of murine skin 28 days after sham surgery (top) as well as hSTSGs (middle) and ADM (bottom) 28 days after implantation. Middle: Curvelet transformation as overlay on histologic image. Red dots indicate the center of fiber segments; green lines indicate the fiber orientation. The small red square indicates the location of the magnified inset. Right: Alignment heatmap. (**E**) Fiber alignment over time. PI, preimplantation; D1 to D28 indicate days after in vivo implantation. (**F**) PCA on CT-FIRE and CurveAlign parameters. (**G**) Immunofluorescent staining of hSTSGs and ADM with overlying murine skin (D14) as well as murine skin after sham surgery for F4/80 and (**H**) CD163. Dotted lines indicate the interface between murine dermis and implanted grafts. White rectangles indicate the location of the magnified images (right). Scale bars, 200 μm in overview images (left) and sham image, and 50 μm in magnified images (right). px, pixels.

To characterize the functional role of macrophages in the cellular response to biologic scaffolds, we subcutaneously implanted either hSTSG-containing viable cells (*16*) or human-derived acellular dermal matrix (ADM) grafts into C57/BL6 [wild-type (WT)] mice (*8*). The grafts themselves and the overlying murine skin were explanted after 1, 3, 7, 14, and 28 days (*n* = 6 to 8 per group) (Fig. 1A). A sham surgery (creation of a subcutaneous pocket without graft implantation) served as a control to account for the impact of the surgical procedure itself (fig. S1A). We found that collagen deposition within both grafts significantly increased over time, indicating that both scaffolds strongly influenced murine collagen production [one-way analysis of variance (ANOVA); **P = 0.006 for day 14 (D14) versus D1; *P = 0.03 for D14 versus D3; *P = 0.02 for D14 versus D7; **P = 0.007 for D28 versus D1 and D3; **P = 0.005 for D28 versus D7] (Fig. 1, B and C). To examine collagen architecture over time, we used the CT-FIRE algorithm to perform single-fiber extraction and analysis in histologic images (*17*) as well as CurveAlign, a curvelet transform–based fibrillar collagen quantification platform to analyze fiber alignment as a surrogate parameter for fibrosis (*18*). While hSTSGs and ADM both showed a significantly higher fiber alignment (*P < 0.05) compared to sham in the early implantation period (before D7), the alignment of hSTSGs decreased after D7 and became similar to murine dermis on D14, while ADM generated a consistently higher alignment than murine dermis throughout the entire implantation period until D28. This chronic, increased alignment indicated progressive fibrosis following ADM implantation. In contrast, long-term implantation of hSTSGs induced a basket weave–like fiber pattern resembling the physiologic dermal collagen architecture of murine skin, with a significantly lower alignment compared to ADM on D28 (one-way ANOVA: D1: ***P = 0.0009 for ADM versus sham and **P = 0.02 for hSTSG versus sham; D3: **P = 0.004 for ADM versus hSTSG, ***P = 0.0002 for hSTSG versus sham, and ****P < 0.0001 for ADM versus sham; D7: *P = 0.03 for hSTSG versus sham and *P = 0.02 for ADM versus sham; D14: **P = 0.001 for ADM versus sham; D28: *P = 0.01 for hSTSG versus ADM) (Fig. 1, D and E, and fig. S1B). This was supported by principal components analysis (PCA) on all output parameters from CT-FIRE and CurveAlign, showing that hSTSG samples, but not ADM samples, clustered more closely together with samples from the sham surgery group (Fig. 1F and fig. S2).

We further performed fractal analysis to compare the histologic architecture between hSTSG and ADM grafts after implantation. Fractal analysis applies nontraditional mathematics to complex patterns that defy understanding with traditional Euclidean geometry (*19*). The overall tissue complexity of hSTSGs, defined by the fractal dimension, was significantly higher at D28 compared to ADM (*P = 0.04), which is in line with a more heterogeneous basket weave–like physiologic skin architecture (fig. S3, A and B).

Because macrophages are the primary cell type that responds to the implantation of foreign bodies (*20*), we examined macrophage infiltration within the scaffolds and surrounding murine tissue on D14, when the collagen architecture of the two grafts began to diverge. We observed a significantly higher number of macrophages around the implanted hSTSGs compared to ADM and sham by immunofluorescent staining for the generic macrophage marker F4/80 (one-way ANOVA: *P = 0.04; Fig. 1G) (*21*). Macrophages recruited to hSTSGs also showed a significantly higher expression of the regenerative M2 macrophage marker CD163 (one-way ANOVA: *P = 0.03; Fig. 1H) (*22*), indicating that the hSTSG microenvironment might induce macrophage recruitment and polarization toward regenerative phenotypes.

### Human cellular elements degrade after murine subcutaneous implantation

To confirm that the identified cells were of murine and not human (i.e., grafted) origin, we performed real-time quantitative polymerase chain reaction (qPCR) for human Alu elements (primate-specific short interspersed elements), as previously described (*23*), on both nonimplanted hSTSGs and hSTSGs explanted after 14 days of subcutaneous implantation. Murine skin was analyzed as a negative control, and live human umbilical vein endothelial cells (HUVECs) were used as a positive control. High levels of human Alu DNA were detected in nonimplanted hSTSGs, which even exceeded those of HUVECs (*P < 0.01), indicating the presence of live human cells within hSTSGs and confirming the findings from previous studies (*16*). After 14 days of subcutaneous implantation, Alu DNA levels in hSTSGs had significantly decreased by several orders of magnitude, indicating that the human cells disappeared and only murine cells were present (fig. S3C).

### hSTSG promotes greater recruitment of circulating cells than ADM

**Fig. 2.**
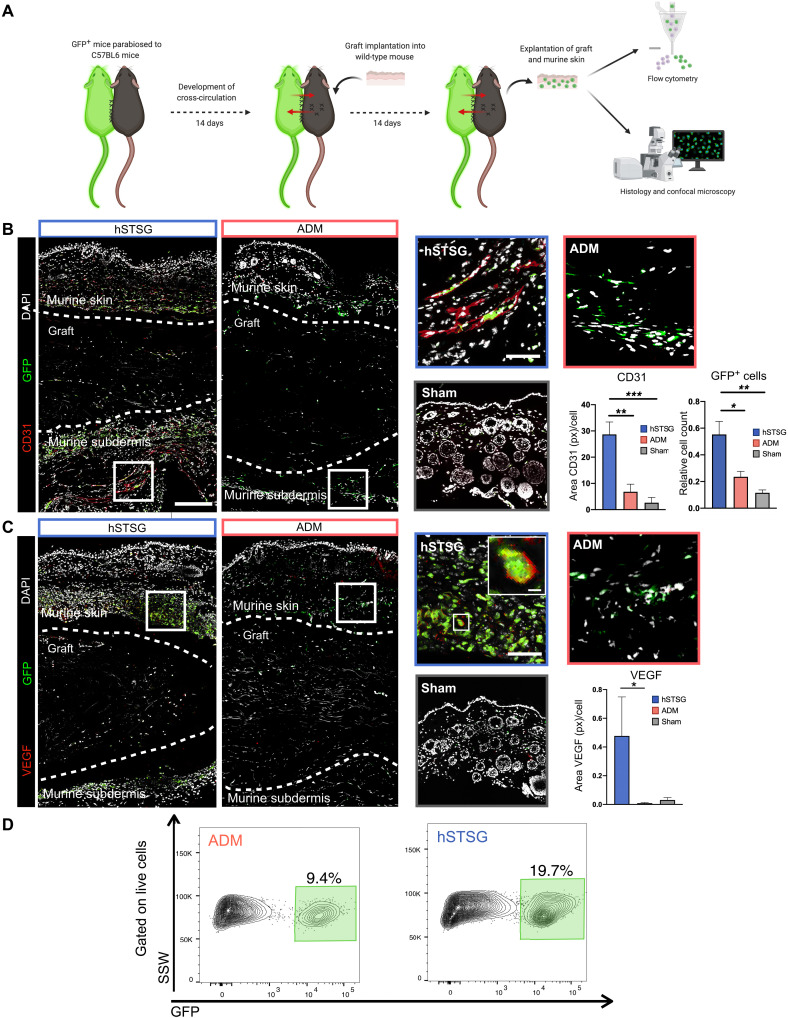
hSTSG promotes strong recruitment of circulating cells compared to ADM. (**A**) Schematic of the parabiosis model (*n* = 5 pairs per group). (**B**) Confocal microscopy of hSTSGs and ADM with overlying murine skin as well as sham tissue showing circulating GFP^+^ cells and immunofluorescent staining for CD31. (**C**) Circulating GFP^+^ cells and immunofluorescent staining for VEGF. (**D**) Flow cytometry analysis of circulating GFP^+^ cells {gating on live [4′,6-diamidino-2-phenylindole (DAPI)–negative] single cells}. Dotted line on histology images indicates the interface between murine skin and graft. White rectangles indicate the location of the magnified images (right). Scale bars, 200 μm in overview images (left) and sham image, 50 μm in magnified images (right), and 20 μm in magnified image showing VEGF- and GFP-expressing circulating cell. SSW, side scatter width.

To examine whether tissue-resident or tissue-circulating cells were recruited to the grafts, we performed parabiosis of green fluorescent protein (GFP)–expressing mice to WT mice and performed either hSTSG or ADM implantation or a sham surgery on the WT mice (Fig. 2A). Confocal laser scanning microscopy of the explanted grafts showed a significantly stronger recruitment of circulating GFP^+^ cells to hSTSGs compared to ADM or sham tissue (one-way ANOVA: relative cell count: *P = 0.01 versus ADM and **P = 0.002 versus sham; Fig. 2B). The circulating cells recruited to hSTSGs showed a higher expression of vascular endothelial growth factor (VEGF) compared to those in ADM and sham tissue [VEGF area (px) per cell: one-way ANOVA: *P = 0.04 for hSTSG versus ADM and *P* = 0.06 versus sham; Fig. 2C], suggesting that hSTSG implantation promotes proangiogenic cell populations. In accordance, we observed a significantly stronger vascularization of the murine tissue surrounding hSTSG compared to ADM and sham tissue [CD31 area (px) per cell: one-way ANOVA: **P = 0.002 for hSTSG versus ADM and ****P = 0.0006 versus sham; Fig. 2B]. Flow cytometry of cells isolated from explanted grafts confirmed a stronger infiltration of GFP^+^ cells in hSTSG versus ADM (19.7% versus 9.4% of all live cells) (Fig. 2D and fig. S4).

### scRNA-seq and flow cytometry reveal heterogeneous macrophage subpopulations infiltrating hSTSGs

**Fig. 3.**
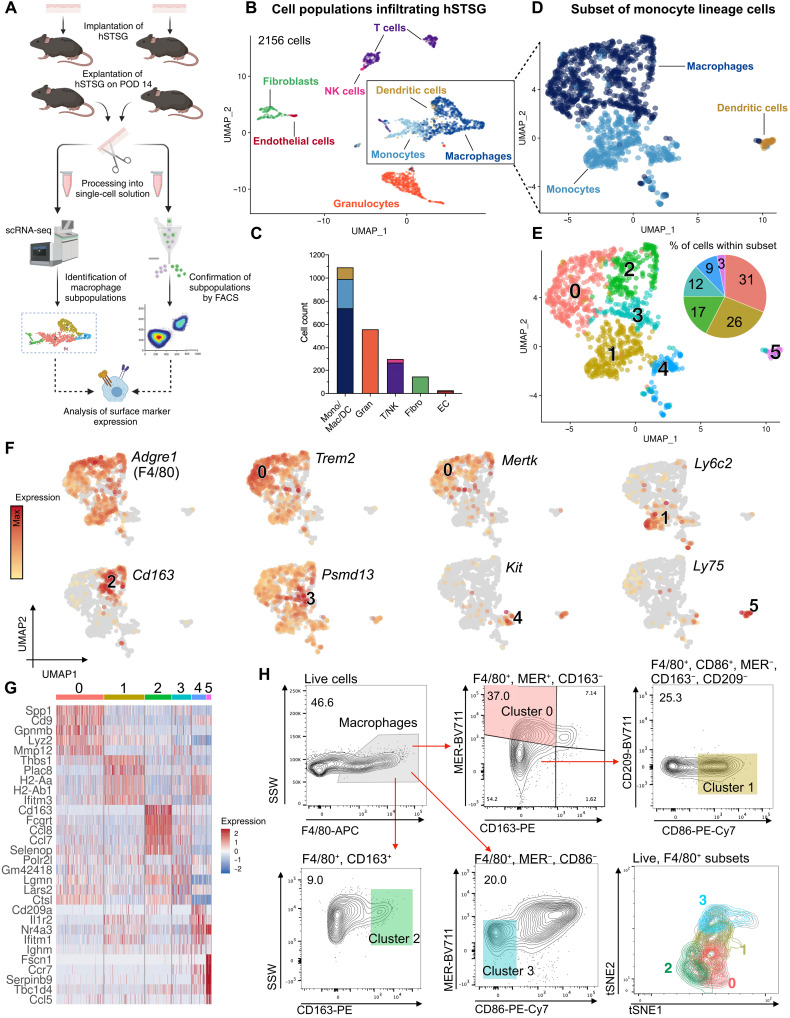
scRNA-seq and flow cytometry reveal heterogeneous macrophage subpopulations infiltrating hSTSGs. (**A**) Experimental overview. (**B**) Cells colored by cell type. (**C**) Bar plot (colored by cell type) indicating the cell number within the populations of (B). Mono, monocytes; Macro, macrophages; Gran, granulocytes; T/NK, T cells and natural killer cells; Fibro, fibroblasts; EC, endothelial cells. (**D**) Subset of monocyte lineage cells. (**E**) Cell clusters of monocyte lineage cells. The pie chart (colored by cluster) shows the relative number of cells within the monocyte lineage subset. (**F**) Feature plots for *Adgre1* (encodes for F4/80) and cluster-defining markers. (**G**) Heatmap of the top five differentially expressed genes per cluster, sorted by average log fold change and ordered by cluster number. (**H**) Flow cytometry gating strategy for cells isolated from hSTSGs after 14 days of implantation (*n* = 5 mice). Single cells were gated for live (DAPI-negative) cells. Macrophages were identified by their expression of F4/80 based on the strong expression of *Adgre1* in monocytes and macrophages. Bottom right panel: tSNE (t-distributed stochastic neighbor embedding) projection for virtual aggregate of flow cytometry data. Macrophage subpopulations were back-gated and are colored by cluster color.

To further understand the mechanisms driving these putatively regenerative cells, we performed scRNA-seq on hSTSGs explanted on D14 using the 10X Genomics Chromium platform (10X Genomics, Pleasanton, CA) (*n* = 5 mice; Fig. 3A). Data for 2156 high-quality cells were embedded into a uniform manifold approximation and projection (UMAP) space using the Seurat package in R (*24*, *25*) and identified as macrophages, monocytes, dendritic cells (DCs), granulocytes, T cells, fibroblasts, and endothelial cells (Fig. 3B and fig. S5A) (*26*, *27*). We then further analyzed the largest cell population (1108 cells), containing monocytes, macrophages, and DCs (Fig. 3, C and D), as a subset and found six distinct cell clusters (labeled 0 to 5) (Fig. 3E). Among these, clusters 0, 2, and 3 were classified as macrophages, expressing the canonical markers *Adgre1* (F4/80) and *Cd68*; clusters 1 and 4 were identified as monocytes; and cluster 5 was classified as DCs (Fig. 3F and fig. S5, B to F).

We identified cluster-defining marker genes and then confirmed them on the protein level using flow cytometric analysis of explanted grafts from a repeated group of mice (*n* = 5; Fig. 3H and fig. S6A). Cluster 0 was the largest macrophage cluster (31% of cells in the subset) and characterized by a strong expression of *Trem2*, *Mertk* (proto-oncogene tyrosine-protein kinase MER), *Cd9*, and *Cd63* and a lack of *Cd163* expression (Fig. 3, F and G, and fig. S5, B and F). Flow cytometry confirmed that F4/80^+^, MER^+^, CD163^−^ cells were the most abundant macrophage subpopulation infiltrating hSTSGs (37%) (Fig. 3H). Cluster 1 cells were identified as Ly6c^high^ monocytes, showing an enrichment of *Ly6c2*, *Plac8*, *Cd86*, and MHCII (major histocompatibility complex class II)–encoding genes (*H2-Aa*, *H2-Ab1*, *H2-DMb1*, and *H2-Eb1*) and depletion of M2 markers *Cd163*, *Mertk*, and *Cd209a* (Fig. 3, F and G, and fig. S5, C and F), and confirmed on the protein level by fluorescence-activated cell sorting (FACS) (26% F4/80^+^, MER^−^, CD163^−^, CD86^+^, CD209^−^ cells) (Fig. 3H). Cluster 2 showed an enrichment of the regenerative macrophage markers *Mrc1* (CD206), *Cd163*, *Lyve1*, and *Retnla*, consistent with a recently described population of perivascular macrophages (Fig. 3, F and G, and fig. S5, D and F) (*28*), and was confirmed by FACS (F4/80^+^, Cd163^+^ cells) (Fig. 3H). Cluster 3 had the least distinct transcriptional profile and appeared to be a transitional state, showing an enrichment of the proteasome gene *Psmd13* and depletion of *Mertk* and *Cd86*; this profile was also confirmed with FACS (F4/80^+^, MER^−^, CD86^−^) (Fig. 3, F to H). Cluster 4 appeared to be a precursor cell population with the potential to differentiate into monocytes or DCs and expressed the hematopoietic stem cell marker *Kit* (proto-oncogene tyrosine-protein kinase Kit), monocyte marker *Ly6c*, and DC lineage markers *Cd209a* (DC-SIGN) and *Flt3* (Fig. 3, F and G, and fig. S5, E and F). Cluster 5 was a transcriptionally distinct cluster of DCs, displaying a high expression of common DC markers *Ly75*, *Flt3*, and *Ccr7* (Fig. 3, F and G, and fig. S5, E and F).

### Trem2^+^ macrophages express proangiogenic and antifibrotic transcriptomic signatures

**Fig. 4.**
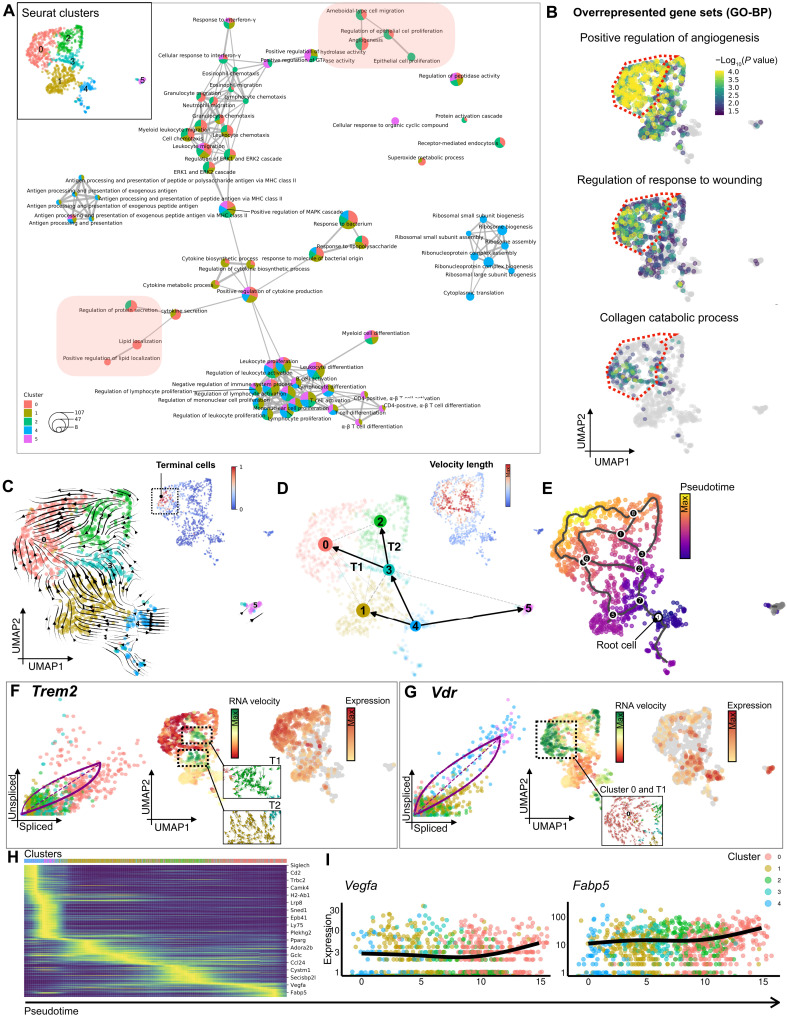
Trem2^+^ macrophages express proangiogenic and antifibrotic transcriptomic signatures. (**A)** Network diagram of enriched gene sets. (**B**) ORA of the indicated gene sets. (**C**) RNA velocities projected onto UMAP embedding. Inset: Terminal cell state within Seurat cluster 0. (**D**) Partition-based graph abstraction quantifies the connectivity of subpopulations; edge weights represent confidence in the presence of connections. T1 and T2: Trajectories leading from cluster 3 to clusters 0 and 2. Inset: Cells colored by length of the velocity vectors as correlate of the differentiation rate. (**E**) Cells colored by pseudotime. Black nodes represent the trajectory branch points; white node represents the root. (**F**) Left: Gene-resolved velocities for Trem2. Middle: Cells colored by RNA velocity; T1 and T2 highlighted by dotted lines; magnified images show velocity vectors of the trajectory cells. Right: Mature (spliced) mRNA expression of Trem2. (**G**) Left: Gene-resolved velocities for *Vdr.* Middle: Cells colored by RNA velocity; cluster 0 highlighted by dotted lines; magnified image shows velocity vectors of cluster 0 cells. Right: Mature (spliced) mRNA expression of *Vdr*. (**H**) Heatmap highlighting genes with high correlation with velocity pseudotime. (**I**) Expression of *Vegfa* and *Fabp5* along pseudotime. Cells are colored by Seurat cluster.

To further characterize the role of these myeloid subpopulations, we constructed a network of enriched gene sets from the Gene Ontology (GO) database (*29*). This analysis indicated that the macrophages in clusters 0 and 2 were enriched for gene sets associated with angiogenesis, cell migration, and epithelial cell proliferation, while the monocytes in cluster 1 were enriched for gene sets related to cytokine biosynthesis and metabolism (Fig. 4A, pie charts showing the relative contribution of each Seurat cluster). The size of the pie chart represents the number of enriched genes. Gene sets enriched in clusters 0 and 2 that indicate regenerative transcriptomic programs are highlighted in red. Inset shows the Seurat clusters of the subset of Fig. 1E. Gene sets associated with lipid metabolism were exclusively enriched in cluster 0, indicating that these cells may represent a recently defined subpopulation of lipid-associated macrophages, which have shown anti-inflammatory properties and protective functions against adipocyte hypertrophy and metabolic disease (Fig. 4A) (*30*). Overrepresentation analysis (ORA) at the single-cell level using GeneTrail 3 (*31*) confirmed a significant enrichment of gene sets for positive regulation of angiogenesis, wound healing, and collagen catabolism in the Trem2^+^ cells in cluster 0, suggesting a proangiogenic and antifibrotic potential (Fig. 4B; dotted lines indicate Seurat cluster 0, which showed the highest Trem2 expression). While higher VEGF expression drives angiogenesis, which is beneficial for wound healing (*32*), previous research has also found profibrotic effects of VEGF under specific conditions (*33*). Thus, the regenerative effect of Trem2^+^ macrophages on graft remodeling is more likely related to their secretion of matrix metalloproteinases (MMPs) such as *Mmp12* and *Mmp13* (fig. S6, B to D), which break down collagen fibers within the grafts to mitigate fibrosis. Summarizing these findings, we hypothesized that implantation of hSTSGs stimulates macrophage polarization toward Trem2^+^ subpopulations that promote collagen remodeling and angiogenesis.

### VD receptor signaling drives transcriptional dynamics of macrophage differentiation

To investigate the transcriptional dynamics of macrophage differentiation, we performed RNA velocity analysis using scVelo (*34*). While comparison of mRNA abundance between cell clusters only captures a static snapshot, RNA velocity analysis predicts the future states of cellular subpopulations by integrating the relative abundance of nascent (unspliced) and mature (spliced) mRNA. Our analysis revealed a differentiation stream originating from cluster 4, which was identified as a root cluster, with a dual commitment toward DCs (cluster 5) and monocytes/macrophages (clusters 1 and 3), matching observations from our differential expression analysis (Fig. 4, C and D, and fig. S6, E to G). The highest differentiation rate, indicated by the velocity vector length (Fig. 4D, inset), was found along two major differentiation trajectories, T1 and T2, indicating that macrophage differentiation from the root cluster was driven along these paths toward the putative regenerative clusters 0 and 2 (Fig. 4D). The Trem2^+^ cells in cluster 0 were identified as the terminal differentiation state (Fig. 4C, inset) and also demonstrated the most advanced progression along pseudotime from the root cells in cluster 4 (Fig. 4E and fig. S6H) (*35*).

**Fig. 5.**
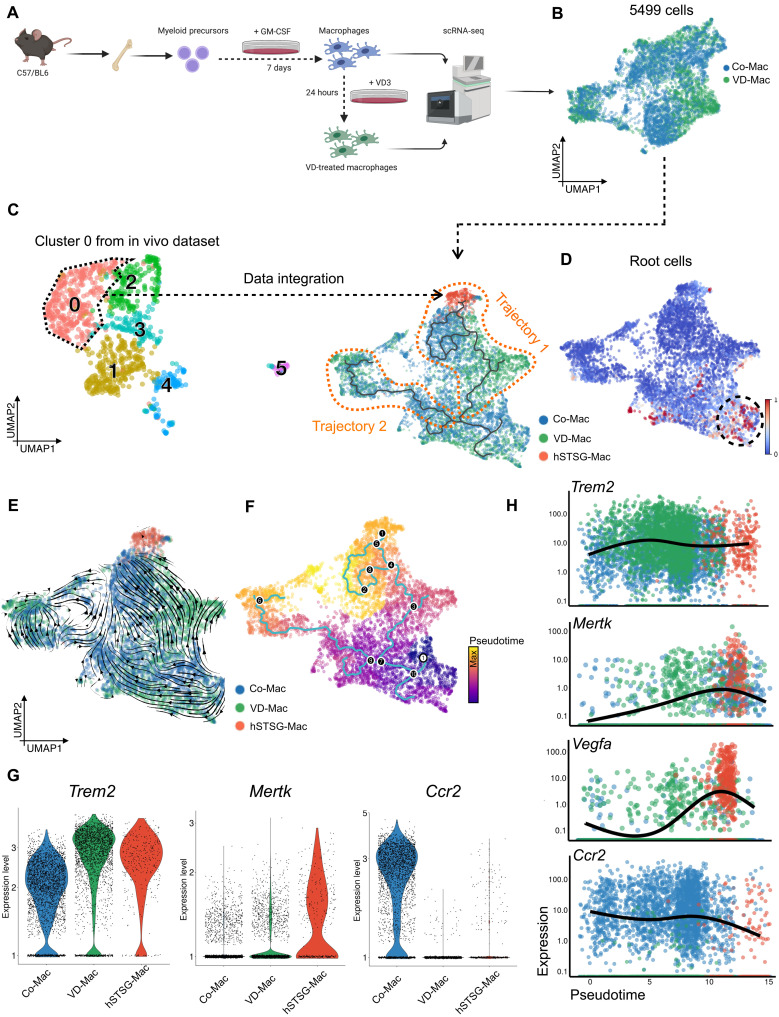
VD3 induces Trem2^+^ macrophage subpopulations in vitro. (**A**) Experimental workflow. BM was isolated from C57/BL6 mice, and cells were differentiated into “M1” macrophages for 7 days in the presence of granulocyte-macrophage colony-stimulating factor (GM-CSF) according to standard protocols (Materials and Methods). The differentiated cells were then either stimulated with 1,25-dihydroxycholecalciferol (active form of VD, VD3) for 24 hours or left untreated, and both populations were analyzed using scRNA-seq. (**B**) UMAP embedding of cells colored by group. Co-Mac, control macrophages; VD-Mac, VD3-treated macrophages. (**C**) Cells from cluster 0 from the in vivo monocyte lineage subset (Fig. 3E) were bioinformatically integrated with data from cultured macrophages. hSTSG-Mac, cluster 0 macrophages (Trem2^+^) from the in vivo dataset. Trajectories (highlighted by orange dotted lines) were determined using Monocle 3. (**D**) Root cells identified by RNA velocity analysis. (**E**) Main gene-averaged flow visualized by velocity streamlines and projected onto the UMAP embedding of the integrated dataset. (**F**) Cells colored by pseudotime using Monocle 3. Black nodes represent the branch points of the trajectories; white node represents the root node. (**G**) Violin plots showing *Trem2*, *Mertk*, and *Ccr2* expression. Cells are colored by experimental group. (**H**) Expression of *Trem2*, *Mertk*, *Vegfa*, and *Ccr2* along pseudotime. Cells are colored by experimental group.

While cluster 0 showed the highest expression of mature, spliced Trem2 mRNA, a particularly strong expression of nascent, unspliced mRNA, characterized by positive velocities, was found along T1 and T2, indicating an induction of Trem2 mRNA transcription (Fig. 4F, the dotted line represents the estimated “steady-state” ratio of unspliced-to-spliced mRNA abundance). A strongly increased transcription rate for the VD receptor–encoding gene, *Vdr*, was found along T1 as well as in cluster 0, shown by an abundance of unspliced *Vdr* mRNA (Fig. 4G). This indicated that activation of the VD receptor potentially drives T1 and the differentiation of macrophages into the regenerative Trem2^+^ subpopulation. Other co-regulated genes (modules) with a high correlation with pseudotime along the main differentiation trajectories included *Lpl* (lipoprotein lipase), a central regulator of lipid metabolism; *Mfge8* [milk fat globule-EGF (epidermal growth factor) factor 8 protein], which diminishes tissue fibrosis and promotes angiogenesis in cutaneous wound healing (*36*, *37*); *Fabp5* (fatty acid–binding protein 5); and *Vegfa*. These genes are consistent with the transcriptional signature of Trem2^+^ lipid–associated macrophages and demonstrate their proangiogenic and antifibrotic phenotypes (Fig. 5, H and I).

### VD3 induces Trem2^+^ macrophage differentiation and clonal proliferation

To confirm that VD receptor signaling drives macrophage differentiation toward Trem2^+^ subpopulations, we analyzed the impact of 1,25-dihydroxycholecalciferol (active form of VD, VD3) on the transcriptional signatures of macrophages in vitro. BM cells were isolated from WT mice, differentiated into proinflammatory “M1” macrophages according to standard culture protocols (*38*), and either stimulated with VD3 for 24 hours or left untreated. The gene expression profiles of both populations were analyzed by scRNA-seq (Fig. 5, A and B). VD3-treated cells showed a notable induction of *Vdr* (VD receptor) and *Cyp24a1* (encoding for VD3 24-hydroxylase, which catalyzes VD3 turnover), confirming a strong transcriptional response to VD3 treatment (fig. S7A). To relate these gene expression patterns to the Trem2^+^ macrophages from our in vivo dataset (cluster 0; Fig. 3E), we integrated both datasets (Fig. 5C and fig. S7B) (*39*) and observed that Trem2 expression in cultured macrophages treated with VD3 even exceeded in vivo levels, confirming our hypothesis that VD3 induces Trem2^+^ macrophage subpopulations (Fig. 5G). RNA velocity and pseudotime analysis identified an upward trajectory mainly containing VD3-treated cells (trajectory 1; Fig. 5, D to F) and driven toward the in vivo Trem2^+^ macrophages (cluster i6 in integrated UMAP embedding; fig. S7B). This trajectory could represent the VD signaling–dependent trajectory leading to Trem2^+^ macrophage differentiation and was characterized by increasing *Mertk* and *Vegfa* with decreasing *Ccr2* expression, further confirming transcriptional programs observed in vivo (Fig. 5, G and H).

**Fig. 6.**
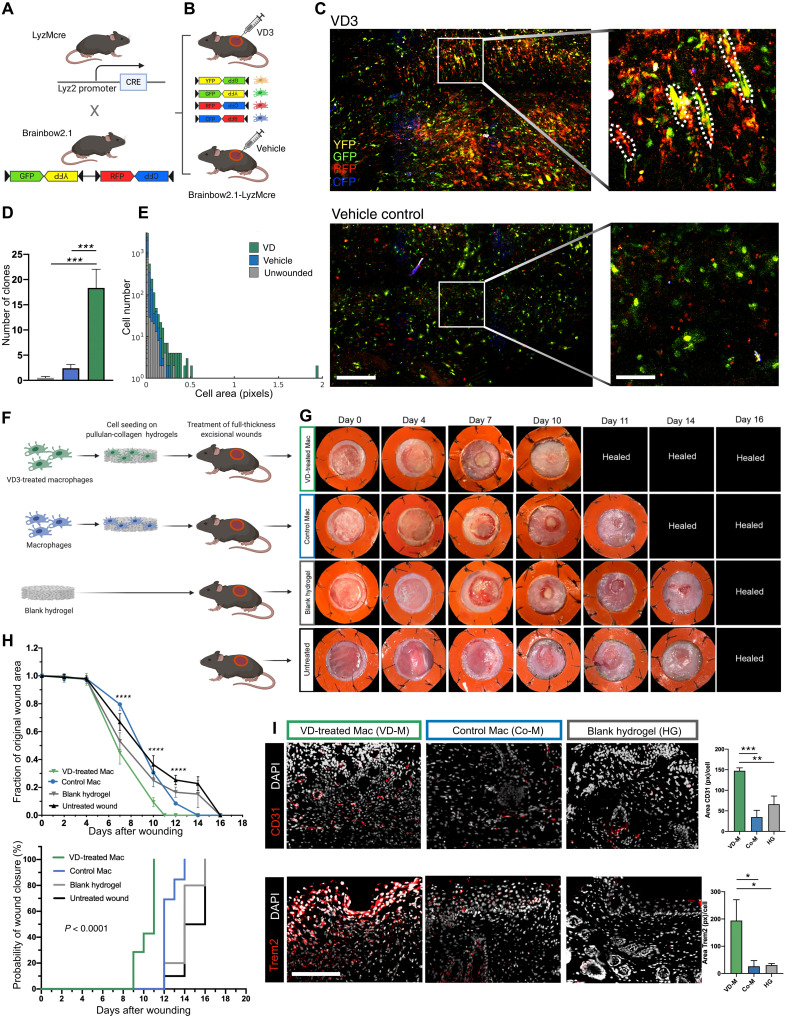
Trem2^+^ macrophage subpopulations accelerate healing of murine full-thickness excisional wounds. (**A**) Breeding schematic for Brainbow2.1-LyzMcre mice. (**B**) Excisional wounds in Brainbow2.1-LyzMcre mice were injected with either 1,25-dihydroxyvitamin D3 (VD3) or a vehicle control (0.9% ethanol in PBS) (*n* = 4 per group). (**C**) Confocal microscopy of the wound bed after VD3 injection (top) and vehicle control (bottom). Scale bars: 100 μm in overview and 20 μm in magnified images. (**D**) Number of clones in wound bed after VD3 injection compared to vehicle injection or unwounded skin. (**E**) Myeloid cell clones within wound beds injected with VD3 showed a higher cell area compared to wounds injected with vehicle control or unwounded skin. (**F**) Schematic of cell seeding experiments (*n* = 5 per group). (**G** and **H**) VD3-treated macrophages significantly accelerated wound healing and led to a significantly reduced wound area at D7, D10, and D12 after wounding compared to control macrophages, untreated hydrogels, and no treatment. (**I**) Immunofluorescent staining of sections from healed wounds from mice treated with VD3-stimulated macrophages (VD-M; explanted on D16 after wounding) showed a significantly higher vascularization and Trem2 expression compared to wounds seeded with control macrophages (Co-M) or blank hydrogels (HG). Scale bar, 200 μm.

We next sought to investigate the effect of VD on myeloid cell proliferation during skin regeneration. We created stented full-thickness excisional wounds on the dorsum of Brainbow2.1-LyzMcre mice, in which *Cre* expression is driven by the myeloid cell–specific *Lyz2* promotor and results in an irreversible recombination of the reporter locus, leading to stochastic expression of one of four fluorescent proteins [GFP, YFP (yellow fluorescent protein), RFP (red fluorescent protein), and CFP (cyan fluorescent protein)] (Fig. 6A and fig. S8, A and B). Following daily VD3 injection into the wound bed, myeloid cells proliferated in a linear, polyclonal manner within the wound and generated a significantly higher number of clones compared to control wounds injected with a vehicle control [0.9% ethanol in phosphate-buffered saline (PBS)] and unwounded skin (one-way ANOVA: ***P = 0.0001 versus control wound and ***P = 0.0002 versus unwounded skin) (Fig. 6, B to E, and fig. S8C). Together, these findings show that VD signaling drives both myeloid differentiation into Trem2^+^ phenotypes and clonal myeloid cell proliferation during wound repair.

### Trem2^+^ macrophage subpopulations accelerate healing of murine full-thickness excisional wounds

To apply these findings toward clinically translatable outcomes, we investigated the capacity of VD3-induced Trem2^+^ macrophages to promote wound healing in vivo. We delivered BM-derived macrophages to stented full-thickness excisional wounds on WT mice using a soft pullulan-collagen hydrogel, previously developed by our group to effectively deliver cell therapies in both small- and large-animal wound models (*40*–*42*). Wounds were either left untreated, treated with blank hydrogels (HG), or treated with hydrogels seeded with VD3-stimulated macrophages (VD-M) or untreated control macrophages (Co-M) (Fig. 6F). VD-M significantly accelerated wound healing and led to a reduced wound area after D7 compared to all other groups (two-way ANOVA: D7 and D10: ****P = 0.0001 versus Co-M, HG, and no treatment; D12: ****P = 0.0001 versus Co-M and no treatment and *P = 0.02 versus HG). Wounds treated with VD-M fully healed by D11, whereas Co-M wounds healed by D14 and HG treated wounds as well as untreated wounds healed by D16 (Fig. 6, G and H). Healed wounds treated with VD-M and explanted on D16 showed significantly higher vascularization (CD31 area in pixels per cell: one-way ANOVA: ***P = 0.0002 for VD-M versus Co-M and **P = 0.002 for VD-M versus HG) and Trem2 expression compared to tissue from both Co-M– and HG-treated wounds. Furthermore, we found that healed wounds treated with VD-M showed a significantly decreased collagen fiber alignment compared to Co-M– and HG-treated wounds. These findings confirmed that Trem2^+^ macrophages induced by VD3 promote angiogenesis, accelerate wound healing, and decrease tissue fibrosis during wound repair (Fig. 6I and fig. S9).

To further confirm that Trem2 is responsible for restoring a physiologic collagen architecture after hSTSG implantation, we investigated how deletion of Trem2 would affect tissue fibrosis after implantation of hSTSGs. hSTSGs were subcutaneously implanted into homozygous Trem2 knockout (KO) mice for 28 days, and the collagen fiber alignment of the explanted xenograft tissue was analyzed and compared to findings from WT mice (Fig. 1D). Confirming our findings, deletion of Trem2 resulted in significantly higher fiber alignment in the grafts (fig. S10), demonstrating that Trem2 is an essential mediator of regenerative healing that is necessary to prevent fibrosis after xenogeneic skin graft implantation and during healing.

## DISCUSSION

The innate immune system plays a critical role in the response to traumatic injury by clearing damaged cells and initiating the tissue repair process, which usually results in scar formation (*43*–*45*). Macrophages are a central component of the innate immune response and are critically involved in all phases of the injury response, from initial inflammation to tissue remodeling. Previous studies have investigated the innate immune response to foreign antigens as a first-line defense mechanism contributing to inflammation and graft rejection (*46*, *47*). Accordingly, the beneficial effects of xenogeneic and allogeneic grafts on wound healing have so far been primarily attributed to their structural matrix component and not to cellular elements within the grafts (*2*–*4*). Our findings indicate that xenogeneic cells within transplanted grafts tune the innate immune response and induce reparative myeloid cells, leading to a beneficial remodeling of their collagenous matrix and surrounding host tissue that improves wound healing.

Using scRNA-seq, we showed that xenogeneic skin grafts induce myeloid cell differentiation toward reparative Trem2^+^ subpopulations. Trem2 is a transmembrane receptor of the immunoglobulin family, which binds anionic ligands such as phospholipids, sulfatides, and DNA. The disruption of cell membranes due to traumatic injury or apoptosis leads to an increase in free DNA and extracellular exposure of phospholipids, which could serve as a trigger for Trem2 receptor-ligand interactions (*48*). Using qPCR of primate-specific DNA elements, we show that human cells within implanted xenogeneic grafts are cleared by the host over time. It seems likely that the resulting abundance of free foreign cellular elements provides a strong stimulus for macrophage differentiation into reparative Trem2^+^ phenotypes. The beneficial impact of local xenogeneic cell application via hSTSGs on wound healing is in line with our previous work, showing that systemic infusion of human MSCs (mesenchymal stromal cells) promotes murine wound healing and macrophage polarization toward regenerative phenotypes within the wound (*49*). The observed regenerative effects resulting from degrading xenogeneic cells may be unique to skin transplantation and not translatable to xenotransplantation of other organs, which require long-term viability of donor cells to ensure functional organ integrity.

Multiple studies have reported on the anti-inflammatory properties of VD and its beneficial effect on wound healing (*50*, *51*), and Vdr-KO models have highlighted the importance of VD on macrophage biology (*52*). However, the role of VD on subsequent macrophage differentiation has remained incompletely understood. Previous studies have identified a connection between VD signaling and Trem2 expression in keratinocytes and myeloid cells of bronchoalveolar lavage fluid (*53*, *54*). Building upon those studies, our findings establish a direct relationship between VD and Trem2 signaling with translational applications. We show that VD3 may be used to induce regenerative Trem2^+^ macrophages in vitro. Future clinicians could use these techniques to easily induce reparative cell types for cell delivery to mediate antifibrotic effects during wound healing.

Trem2^+^ macrophages have previously been shown to be involved in lipid metabolism and the regulation of hair follicle stem cell activity, pointing to an important role in skin regeneration (*55*). Moreover, the regenerative action of Trem2^+^ macrophages has been previously corroborated in other organ systems, such as during colonic mucosal healing or liver regeneration where Trem2^+^ macrophages promote regenerative endothelial cell differentiation (*56*, *57*). Variants in the Trem2 locus are associated with early-onset Alzheimer’s disease in humans, related to a reduced clearance of extracellular amyloid β–containing plaques due to an impaired function of microglia within the brain (*58*, *59*). In mouse models of Alzheimer’s disease, enhancement of Trem2 signaling using agonistic antibodies has recently shown beneficial effects on cognitive function through attenuation of neuroinflammation and improved plaque clearance within the brain (*60*, *61*). The accelerated healing rates after enrichment of the wound bed with Trem2^+^ macrophages that we observed in our study might be due to a more efficient phagocytosis of dead cells and debris within the wound. Moreover, proangiogenic growth factors secreted by Trem2^+^ macrophages may improve vascularization and oxygen supply required for wound healing.

A limitation of our study is related to the fact that rodent models do not exactly recapitulate human wound healing; therefore, future studies are needed to validate our findings in porcine wound models, which more closely mimic human biomechanics and skin physiology (*42*, *62*, *63*). While our study demonstrates a strong regenerative effect of Trem2^+^ macrophages on wound healing, we have investigated only one dosing regimen of 500,000 cells applied weekly to the wound bed. Future studies investigating different dosages and dosing intervals are needed to maximize the therapeutic potential of our novel cell-based therapy for wound healing before clinical translation can be achieved.

Our study uncovers the molecular background driving the long observed beneficial effects of unmatched skin transplantation on wound healing. We demonstrate the preclinical therapeutic potential of Trem2^+^ macrophages to mitigate fibrosis and promote wound healing. We show that regenerative Trem2^+^ macrophages can be externally derived from BM cells in vitro using VD3 and then exogenously applied to wounds as a cell-based therapy.

Our approach to induce Trem2^+^ macrophages and deliver them to the wound bed using hydrogels provides a novel, effective strategy with a high potential for clinical translation. In the clinic, myeloid cells can be readily obtained in high quantities from the peripheral blood using clinically established cell apheresis and culture protocols (*64*), stimulated to differentiate into Trem2 phenotypes with VD3, and easily integrated with the current standard of care to improve wound healing after injury.

## MATERIALS AND METHODS

### Study design

The goal of our study was to investigate the impact of clinically used human biologic scaffolds on the innate immune response and soft tissue remodeling in a murine model over time. We chose a murine subcutaneous implantation model as this allowed us to interrogate the host response and specific microenvironment created by the implanted xenografts in a protected space without any potential external influence. scRNA-seq was used to investigate the subpopulations of myeloid cells emerging after hSTSG implantation and the molecular mechanisms driving their differentiation. To confirm subpopulation-defining markers on the protein level, we used flow cytometry on cells isolated from a repeated group of mice. The impact of VD signaling on clonal proliferation of myeloid cells during wound repair was confirmed using myeloid cell–specific Cre-mediated recombination in a multicolor reporter mouse model. To translate our findings for potential clinical applications, we developed an approach for induction of regenerative Trem2^+^ macrophages in vitro by treating BM-derived macrophages with VD3 and seeded these cells on collagen-pullulan hydrogels. We have previously demonstrated that this technique preserves cell viability and enables efficient cell delivery to wounds (*41*). The capacity of Trem2^+^ macrophages to improve wound healing was investigated in a splinted full-thickness excisional wound model that limits the contraction of the murine panniculus carnosus muscle to recapitulate human physiological wound healing and allows murine wounds to heal by granulation tissue formation and re-epithelialization (*63*).

### Animals

All experiments were performed in accordance with Stanford University Institutional Animal Care and Use Committees and the National Institutes of Health (NIH) *Guide for the Care and Use of Laboratory Animals*. The study was approved by the Administrative Panel on Laboratory Animal Care (APLAC) at Stanford University (APLAC protocol number: 12080). The following mouse strains were obtained from the Jackson Laboratory (Bar Harbor, ME): C57BL/6J (WT), C57BL/6-Tg(CAG-EGFP)1Osb/J, Lyz2^tm1(cre)Ifo^/J (LyzMcre), R26R-Brainbow2.1, and C57BL/6J-*Trem2^em2Adiuj^*/J (Trem2 KO mice). To breed Brainbow2.1-LyzMcre mice, LyzMcre mice, which express Cre recombinase specifically in myeloid cells, were crossed with mice expressing a floxed transgenic four-color reporter construct (Brainbow2.1) at the *Rosa26* locus (R26R-Brainbow2.1). All animal surgeries were performed under inhalation anesthesia with isoflurane (Henry Schein Animal Health) at a concentration of 1 to 2% in oxygen at 3 liters/min. The mice were placed in prone position, and dorsal fur was shaved. Skin was disinfected with betadine solution followed by 70% ethanol three times.

### Xenograft implantation model

For implantation of hSTSGs and ADM, incisions of 1 cm were created on the dorsa of C57BL/6J (WT) mice (Jackson Laboratory, Bar Harbor, ME), and subcutaneous pockets were created by sharp dissection above the muscle fascia. hSTSGs (TheraSkin, Misonix Inc., Farmingdale, NY) and ADM (AlloDerm, Allergan, Dublin, Ireland) were cut into 1 cm by 1 cm pieces and placed into the pockets, which were closed with 6-0 nylon sutures (Ethilon, Ethicon, Somerville, NJ). Sham surgeries were performed by creating pockets without graft implantation on a separate group of mice (*n* = 6) as controls. The grafts and overlaying murine skin were explanted after 1, 3, 7, 14, and 28 days and processed for analysis (*n* = 6 to 8 per each group for ADM, hSTSG, and sham surgeries).

### Parabiosis

Parabiosis was performed as previously described (*65*). Briefly, the corresponding flanks of GFP^+^ [C57BL/6-Tg(CAG-EGFP)1Osb/J] and C57BL6/J mice (WT) were shaved and disinfected with Betadine solution and 70% ethanol three times. Matching skin incisions were made from the olecranon to the knee joint of each mouse. The skin edges were undermined to create skin flaps of 1 cm width. The 6-0 nylon sutures (Ethilon) were used to approximate the dorsal and ventral edges of the skin flaps, and skin staples were used to close the longitudinal incisions. Buprenorphine was used for analgesia by subcutaneous injection every 8 to 12 hours for 48 hours after operation. Mice were monitored daily until the end of the experiment. After 14 days, peripheral blood chimerism was confirmed using fluorescent microscopy of the tail vein blood, before subcutaneous implantation of the grafts into the WT mice as described above. The grafts and the overlaying murine dermis were explanted from the parabiosed mice 14 days after implantation (*n* = 5 per group).

### Splinted excisional wound model

Splinted full-thickness excisional wounds were created as previously described by Galiano *et al*. (*63*). In this model, a silicone ring is sutured around the wound margin to stent the skin, mimicking human physiological wound healing by limiting the contraction of the murine panniculus carnosus muscle and allowing the wounds to heal by granulation tissue formation and re-epithelialization (*63*). Two full-thickness dermal wounds of 6 mm diameter were created on the dorsum of each mouse (C57BL/6J) using biopsy punches. A silicone ring was fixed to the dorsal skin using an adhesive glue (Vetbond, 3M, Saint Paul, MN) and eight interrupted 6-0 nylon sutures placed around the outer edge of the ring to prevent wound contraction. The wounds were treated with collagen-pullulan hydrogels or left untreated and covered with sterile dressings. Digital photographs were taken at the time of surgery and during every dressing change until the time of wound closure. For the hydrogel treatment study, wounds were harvested on D16 after wounding, when the wounds in all groups had healed.

Wounds in Brainbow2.1-LyzM-Cre mice were injected daily with either 100 ng of 1,25-dihydroxyvitamin D3 (dissolved in ethanol and further diluted to 0.9% in PBS) or a vehicle control (0.9% ethanol in PBS). Wounds were harvested on D5 for analysis of clonal macrophage proliferation (*n* = 5 per group).

### Histologic analysis of collagen content

Explanted tissue was fixed in 4% paraformaldehyde in PBS for 24 hours. For Masson’s trichrome staining, tissue was dehydrated in a graded ethanol series and embedded into paraffin. Tissue sections were deparaffinized and rehydrated through graded ethanol series. Masson’s trichrome staining (Sigma-Aldrich) was performed according to the manufacturer’s recommendations and imaged using bright-field microscopy. We then implemented an algorithm in MATLAB to automatically deconvolve the color information of each trichrome image (*66*). We determined a color matrix based on the stain-specific RGB light absorption of these samples

$$C=[\begin{array}{ccc} 0.7995107 & 0.5914 & 0.1053\\ 0.1000 & 0.7374 & 0.6680\\ 0.5923 & 0.3264 & 0.7366 \end{array}]$$

The top two rows correspond to stain-specific RGB values of trichrome red and blue, respectively, and the columns represent the normalized vector values in the red, green, and blue channels. This algorithm allows for a robust and flexible method for objective immunohistochemical analysis of samples stained with up to three different colors.

### Immunofluorescent staining and confocal laser scanning microscopy

After fixation, the tissue was dehydrated in 30% sucrose in PBS for at least 48 hours at 4°C. Tissue was then incubated in optimal cutting temperature compound (O.C.T., TissueTek, Sakura Finetek, Torrance, CA) for 24 hours at 4°C and then cryo-embedded in tissue molds on dry ice. Frozen sections were performed at 7 μm thickness on a cryostat. Antigen retrieval was performed using 0.01 M sodium citrate buffer in PBS (Abcam, Cambridge, MA), followed by blocking for 2 hours in 5% goat serum (Invitrogen, Waltham, MA) in PBS. Sections were then incubated in anti-F4/80 antibody (ab6640, Abcam), anti-CD31 antibody (ab28364, Abcam), anti-MMP13 antibody (ab39012, Abcam), or anti-VEGFA antibody (PA1-21796, Thermo Fisher Scientific) overnight at 4°C. Secondary antibodies were applied for 1 hour at room temperature [Goat anti-Rabbit IgG (H+L) Highly Cross-Adsorbed Secondary Antibody, Alexa Fluor Plus 488; Goat anti-Rat IgG (H+L) Cross-Adsorbed Secondary Antibody, Alexa Fluor 647; Goat anti-Rabbit IgG (H+L) Highly Cross-Adsorbed Secondary Antibody, Alexa Fluor Plus 647]. Imaging was performed on a Zeiss LSM 880 confocal laser scanning microscope at the Cell Sciences Imaging Facility at Stanford University. To obtain high-resolution images of tissue samples, multiple images of ×25 magnification were acquired using automatic tile scanning. Individual images were stitched together during acquisition using the ZEN Black software (Zeiss, Oberkochen, Germany).

### Quantification of immunofluorescent staining

Immunofluorescent staining was quantified using a code written in MATLAB adapted from previous image analysis studies by one of the authors (K.C.) (*67*). Briefly, confocal images were separated into their RGB channels and converted to binary to determine the area covered by each color channel. 4′,6-Diamidino-2-phenylindole (DAPI) stain, corresponding to the blue channel, was converted to binary using imbinarize and an image-specific, automated threshold determined from the function graythresh to optimize the number of DAPI cells counted within the image. For the red and green channels, which corresponded to the actual protein stains, we converted these images to binary with a consistent threshold of ~0.3 for all images of the same stain. This consistent threshold insured that our automated quantification of the stain area would be unbiased. The area of red and green stains was then normalized by dividing by the number of cells, which we calculated as the number of DAPI nuclei above size threshold of 15 pixels.

To quantify GFP^+^ cells in histologic images, each GFP^+^ signal is analyzed and counted from separate objects (cells) in MATLAB using the bwlabeln and regionprops functions as previously published by the authors (*67*). Briefly, we converted the immunofluorescent images into binary images and then counted the number of separate blue (DAPI) and green (GFP^+^) objects to determine the relative number of GFP^+^ cells normalized to all (DAPI-positive) cells (relative cell count).

For clonal analysis, a size of 2000 pixels was chosen to delineate between either singular cells or clones. This threshold was chosen by visually inspecting images and counting an average size of inspected clones.

### Quantification of collagen fiber architecture

Analysis of fiber alignment of the murine dermis from the sham surgery group as well as the ADM and hSTSG grafts was performed on images (×25 magnification) of Masson’s trichrome–stained tissue sections. Collagen fiber quantification was performed using CT-FIRE and CurveAlign, an open-source software package for automatic segmentation and quantification of individual collagen fiber (http://loci.wisc.edu/software/ctfire) (*17*). Briefly, CurveAlign quantifies all fiber angles and strength of alignment within an image, while CT-FIRE analyzes individual fiber metrics such as length, width, angle, and curvature. It can also extract other variables such as localized fiber density and the spatial relationship between fiber and the associated boundary. The average fiber parameters for each mouse were used for statistical analysis. Statistical analysis was performed in Prism8 (GraphPad, San Diego, CA) using Student’s *t* test or one-way ANOVA with Tukey’s multiple comparisons test. The Rayleigh test was used to assess the circular distribution of the calculated vectors of alignment. Data are presented as means ± SEM. *P* values < 0.05 were considered statistically significant.

PCA was performed on all output parameters of CT-FIRE and CurveAlign (overall alignment, nearest neighbor alignment, nearest fiber distance, fiber width, and fiber length) with centering to mean zero and scaling to unit variance using the prcomp function in R. Plots were generated with 95% confidence intervals using the ggbiplot and pca3d packages.

### Flow cytometry

Analysis of murine cells isolated from explanted grafts was performed according to published protocols for flow cytometry on murine tissue (*68*). Briefly, hSTSGs and ADM were explanted on D14 from the parabiosis and nonparabiosis implantation models and then microdissected and incubated in serum-free Dulbecco’s modified Eagle’s medium with 240 U of collagenase IV per milliliter for 1 hour at 37°C in a rotating oven. Digested tissue was filtered, centrifuged, and stained with BV711 rat anti-mouse Mer antibody, phycoerythrin (PE)/Cy7 rat anti-mouse CD86 antibody, PE rat anti-mouse CD163 antibody, BV605 rat anti-mouse CD209a antibody, and allophycocyanin (APC) rat anti-mouse F4/80 antibody (BioLegend, San Diego, CA). DAPI was used to stain dead cells. Flow cytometry was performed on BD FACSAria (Becton Dickinson, San Jose, CA), and data were analyzed using FlowJo (Becton Dickinson, San Jose, CA).

### Real-time qPCR

Alu-qPCR was performed using a protocol and primers (101 forward and 206 reverse) previously developed by Funakoshi *et al.* (*23*). The total reaction volume was 20 μl and contained 10 μl of TaqMan Universal Master Mix II, no UNG (uracil-N-glycoslyase) (Thermo Fisher Scientific), 0.2 μM forward and reverse primers, 0.25 μM hydrolysis probe, and the appropriate amount of genomic DNA (40 ng per reaction) on an Applied Biosystems 7900 instrument (Thermo Fisher Scientific). qPCR conditions were 1 cycle of 95°C for 10 min, followed by 50 cycles of 95°C for 15 s, 56°C for 30 s, and 72°C for 30 s. All samples were run as triplicates. β-Actin was used as a reference gene. Fold changes were calculated using the 2^−ΔΔ^ method (*69*). qPCR for genotype confirmation of transgenic Brainbow2.1-Lyz2M-Cre mice was performed from DNA isolated from the tail tissue by Transnetyx (Cordova, TN).

### scRNA-seq data processing, normalization, and cell cluster identification

Base calls were converted to reads using the Cell Ranger (10X Genomics, Pleasanton, CA, USA; version 3.1) implementation mkfastq and then aligned against the GRCh38 v3.0.0 (human) genome using Cell Ranger’s count function with SC3Pv3 chemistry and 5000 expected cells per sample. Cell barcodes representative of quality cells were delineated from barcodes of apoptotic cells or background RNA based on a threshold of having at least 300 unique transcripts profiled, less than 100,000 total transcripts, and less than 10% of their transcriptome of mitochondrial origin. Unique molecular identifiers (UMIs) from each cell barcode were retained for all downstream analysis. Raw UMI counts were normalized with a scale factor of 10,000 UMIs per cell and subsequently natural log-transformed with a pseudocount of 1 using the R package Seurat (version 3.1.1) (*25*). Aggregated data were then evaluated using UMAP analysis over the first 15 principal components (*24*). Cell annotations were ascribed using the SingleR package (version 3.11) against the ImmGen database (*26*, *27*) and confirmed by expression analysis of specific cell type markers. Louvain clustering was performed using a resolution of 0.5 and 15 nearest neighbors.

### Generation of characteristic subpopulation markers, enrichment, and ORA

Cell type markers were generated using Seurat’s native FindMarkers function with a log fold change threshold of 0.25 using the ROC (receiver operating characteristic) to assign predictive power to each gene. The 50 most highly ranked genes from this analysis for each cluster were used to perform ORA using clusterProfiler (version 3.1.1). Using GeneTrail 3, an ORA was performed for each cell using the 500 most expressed protein-coding genes on the gene sets of the Gene Ontology (*31*). *P* values were adjusted using the Benjamini-Hochberg procedure, and gene sets were required to have between 2 and 1000 genes. Stacked violin plots were generated using the Scanpy package (*70*).

### Pseudotime analysis

Pseudotime analysis was performed using the Monocle 3 package in R (version 3 0.2.0) (*71*). A principal graph was learned from the reduced dimension space using reversed graph embedding with default parameters. The principalGraphTest function using the Moran’s *I* statistic was used to identify correlated genes on trajectory embedded in the manifold. As indicated by RNA velocity analysis, cluster 4 was chosen as a root of the trajectory.

### RNA velocity analysis

RNA velocity analysis was performed using scVelo (*34*). In contrast to previous steady-state models, which falsely assume that all genes share a common splicing rate, scVelo uses a likelihood-based dynamical model to solve the full transcriptional dynamics of splicing kinetics. Thereby, RNA velocity analysis can be adapted to transient cell states and heterogeneous cellular subpopulations as in our dataset. Partition-based graph abstraction was performed using the sc.tl.paga function in scVelo. To find genes with differentially regulated transcriptional dynamics compared to all other clusters, a Welch *t* test with overestimated variance to be conservative was applied, using the sc.tl.rank_velocity_genes function. Genes were ranked by their likelihood obtained from the dynamical model grouped by Seurat clusters. A pseudotime heatmap was created by plotting the expression of the genes identified by sc.tl.rank_velocity_genes along velocity-inferred pseudotime. The terminal transcriptional states were identified as end points of the velocity-inferred Markov diffusion process.

### Cultivation of BM-derived macrophages and cell seeding

BM isolation and cultivation of cells into macrophages were performed according to previously published protocols (*72*, *73*). Briefly, femurs and tibias were removed from C57/BL6 mice after euthanasia. The epiphyses on both ends of the long bones were removed. The bones were flushed with 5 ml of RPMI to collect the BM. The epiphyses were crushed using a pestle and mortar to isolate the BM. Red blood cells were lysed with ACK (ammonium-chloride-potassium) lysis buffer. Cells were strained and washed in complete medium 2 × 10^6^ per 100-mm dish in 10 ml of RPMI containing 10% fetal bovine serum, penicillin (100 U/ml), streptomycin (0.1 mg/ml), 2 mM glutamine, 50 μM 2-mercaptoethanol, and 200 Units (U) / ml murine granulocyte-macrophage colony-stimulating factor (GM-CSF; PeproTech, Frankfurt, Germany). On D3, 10 ml of complete medium was added to the cultures, and on D6, 10 ml of medium was collected and cells were spun down and resuspended in fresh medium. On D7, half of the cultures were stimulated with 10^−6^ M 1,25 dihydroxy-cholecalciferol (Sigma-Aldrich) for 24 hours. Cells were collected and seeded on pullulan-collagen hydrogels with a diameter of 8 mm at a concentration of 500,000 cells per hydrogel and wound, using a previously described capillary force seeding technique (*41*). We have previously demonstrated that this approach preserves cell viability as well as hydrogel architecture and enables efficient cell delivery (*41*).

### Statistical analysis

Data are shown as means ± SEM and were analyzed using Prism 8 (GraphPad, La Jolla, CA). Two-group comparisons were performed with Student’s *t* test (unpaired and two-tailed). One- or two-way ANOVA, followed by Benajmini-Hochberg post hoc test was performed for comparisons of more than two groups as appropriate. *P <* 0.05 was considered statistically significant. The statistical methods used for scRNA-seq analysis are described in the specific sections above.
