# Supplementary material for: Xenogeneic skin transplantation promotes angiogenesis and tissue regeneration through activated Trem2+ macrophages
Source: Sci Adv. 2021 Dec 1;7(49):eabi4528. doi: 10.1126/sciadv.abi4528 (PMC8635426; doi:10.1126/sciadv.abi4528)
Supplement: Supplementary file 1 — Figs. S1 to S10 [file sciadv.abi4528_sm.pdf]

Science Advances
AAAS

# Supplementary Materials for

## Xenogeneic skin transplantation promotes angiogenesis and tissue regeneration through activated Trem2$^+$ macrophages

Dominic Henn, Kellen Chen, Tobias Fehlmann, Artem A. Trotsyuk, Dharshan Sivaraj, Zeshaan N. Maan, Clark A. Bonham Jr., Janos A. Barrera, Chyna J. Mays, Autumn H. Greco, Sylvia E. Moortgat Illouz, John Qian Lin, Sydney R. Steele, Deshka S. Foster, Jagannath Padmanabhan, Arash Momeni, Dung Nguyen, Derrick C. Wan, Ulrich Kneser, Michael Januszyk, Andreas Keller, Michael T. Longaker, Geoffrey C. Gurtner*

*Corresponding author. Email: ggurtner@stanford.edu

Published 1 December 2021, *Sci. Adv.* **7**, eabi4528 (2021)
DOI: 10.1126/sciadv.abi4528

**This PDF file includes:**

Figs. S1 to S10

**Supplementary Materials**

Fig. S1. Sham group histology and collagen fiber analysis over time

Fig. S2. Principal component analysis (PCA) on CT-FIRE and CurveAlign output

Fig. S3. Fractal analysis and qPCR for human Alu elements

Fig. S4. Flow cytometry pre-gating on cells from parabiosis model

Fig. S5. Cluster defining marker genes in full scRNA-seq dataset and myeloid cell subcluster

Fig. S6. Flow cytometry pre-gating on macrophage subpopulations, scRNAseq analysis

Fig. S7. scRNAseq analysis of bone marrow derived macrophages

Fig. S8. Genotype confirmation of Brainbow 2.1-LyzMcre mice and histology of unwounded skin

Fig. S9. Collagen fiber analysis in wounds treated with Trem2+ macrophages, control macrophages and blank hydrogels

Fig. S10. Collagen fiber analysis in Trem2-KO model

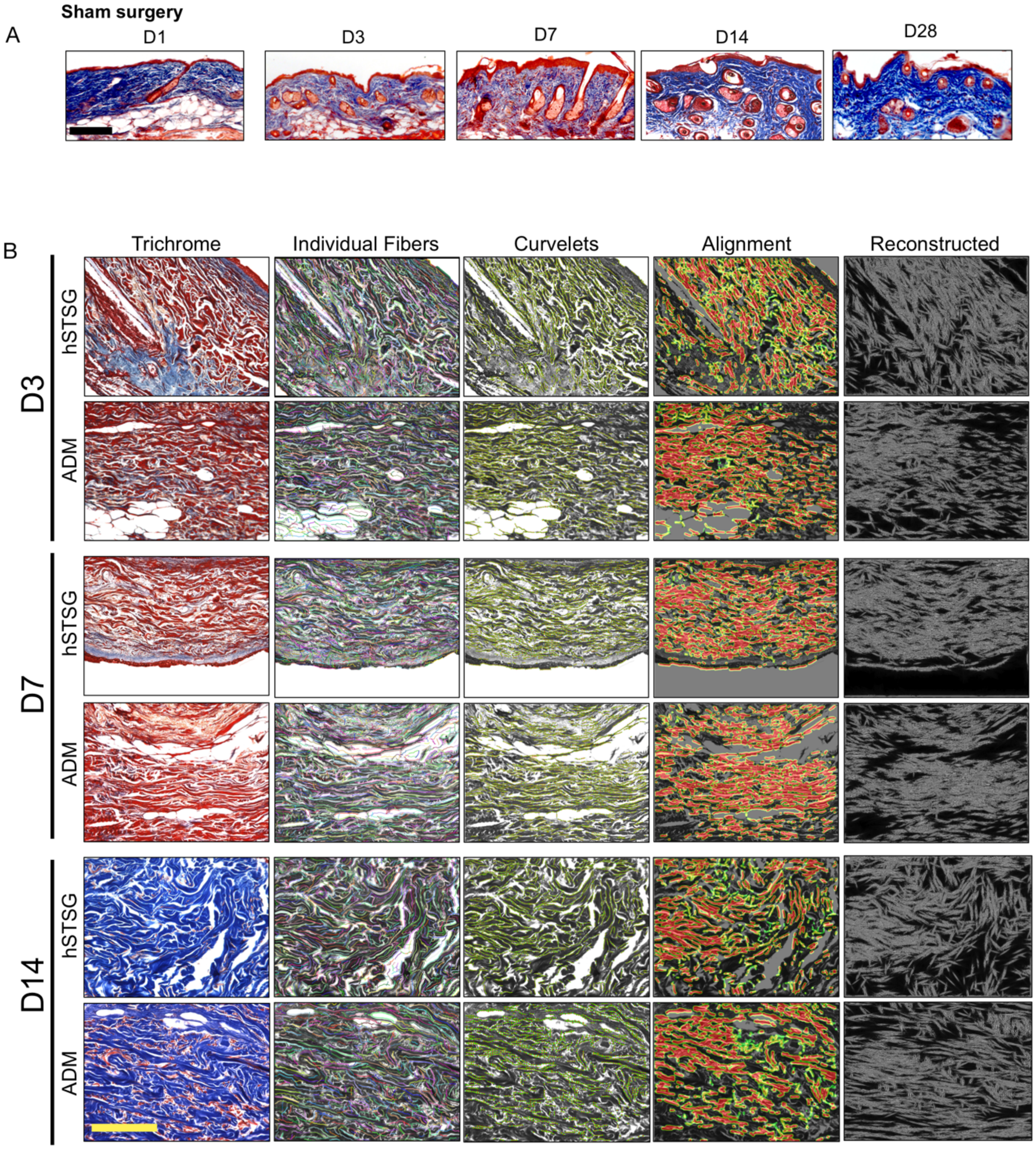

Sham surgery
A
D1
D3
D7
D14
D28
B
Trichrome
Individual Fibers
Curvelets
Alignment
Reconstructed
D3
hSTSG
ADM
D7
hSTSG
ADM
D14
hSTSG
ADM

**Fig. S1.**

**(A)** Masson's trichrome staining of tissue sections from the sham group explanted on days 1, 3, 7, 14, and 28 post-surgery (D1 – D28). (**B)** Far left: Magnified image of collagen architecture of hSTSG and ADM (Masson's trichrome staining). Left: individual fibers identified with CT-FIRE. Middle: curvelet transformation as overlay on histologic image, red dots to indicate the center of fiber segments, green lines indicate the fiber orientation at that point. Right: heatmap of alignment with red indicating the regions with the most aligned fiber angles; far right: image reconstructed using CurveAlign; scale bars: 200µm. hSTSG = human split-thickness skin graft, ADM = acellular dermal matrix graft.

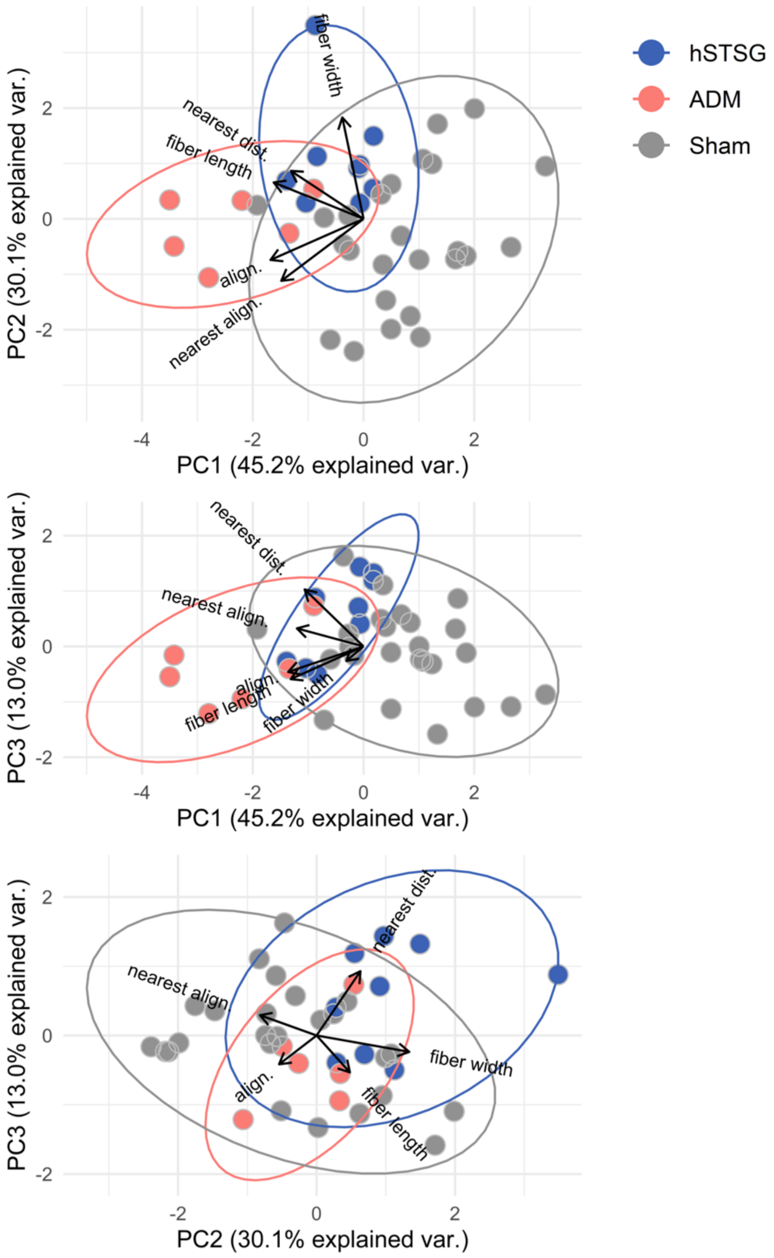
hSTSG
ADM
Sham
PC1 (45.2% explained var.)
PC2 (30.1% explained var.)
PC3 (13.0% explained var.)
fiber width
nearest dist.
fiber length
align.
nearest align.
-4
-2
0
2

**Fig. S2.**

Principal component analysis (PCA) on output parameters from CT-FIRE and CurveAlign analysis of collagen fibers from hSTSG, ADM (28 days post implantation) and skin mice with sham surgery 28 days post surgery. align = alignment, dist = distance, hSTSG = human split-thickness skin graft, ADM = acellular dermal matrix graft.

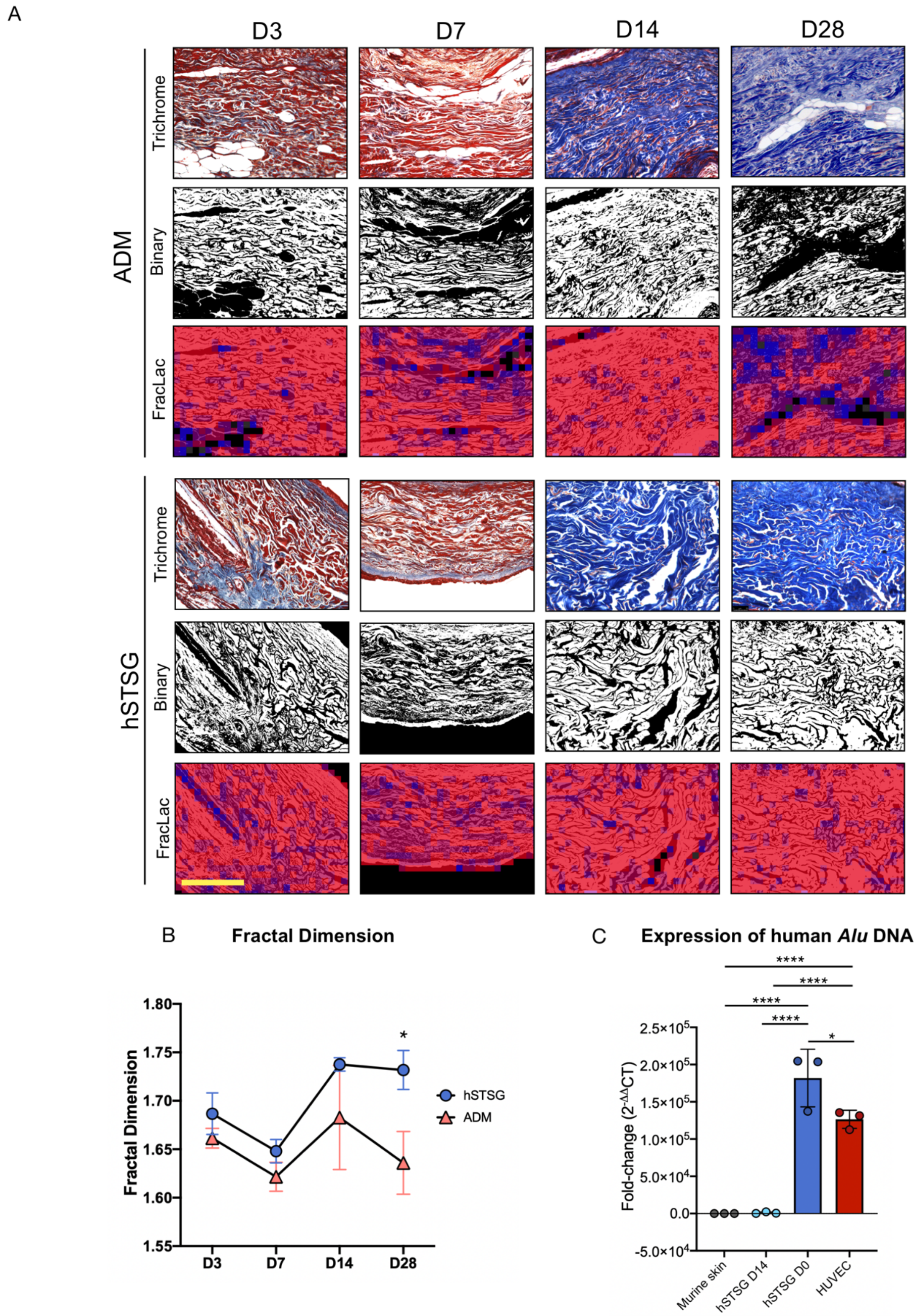
A
D3
D7
D14
D28
ADM
Trichrome
Binary
FracLac
hSTSG
Trichrome
Binary
FracLac
B
Fractal Dimension
Fractal Dimension
1.80
1.75
1.70
1.65
1.60
1.55
D3
D7
D14
D28
*
hSTSG
ADM
C
Expression of human *Alu* DNA
Fold-change ($2^{-\Delta\Delta CT}$)
$2.5\times10^5$
$2.0\times10^5$
$1.5\times10^5$
$1.0\times10^5$
$5.0\times10^4$
0.0
$-5.0\times10^4$
****
****
****
****
*
Murine skin
hSTSG D14
hSTSG D0
HUVEC

**Fig. S3.**

(**A** and **B**) Fractal analysis comparing the histologic architecture between hSTSG and ADM. Two-way ANOVA (n = 5 per group): *P= 0.04; scale bar: 200 µm. (**C**) Real-time PCR (qPCR) for human Alu elements in non-implanted hSTSG (hSTSG D0) and hSTSG explanted after 14 days of subcutaneous implantation into wild-type mice (hSTSG D14). Native murine skin was analyzed as a negative control and live human umbilical vein endothelial cells (HUVEC) were used as a positive control. The HUVEC group represents 3 different cell culture plates. All samples were analyzed as triplicates. Data is shown as fold-change computed by $2^{-\Delta\Delta}$method (*62*); One-way ANOVA (n = 3 per group): * P = 0.01; **** P < 0.0001, hSTSG = human split-thickness skin graft, ADM = acellular dermal matrix graft.

hSTSG

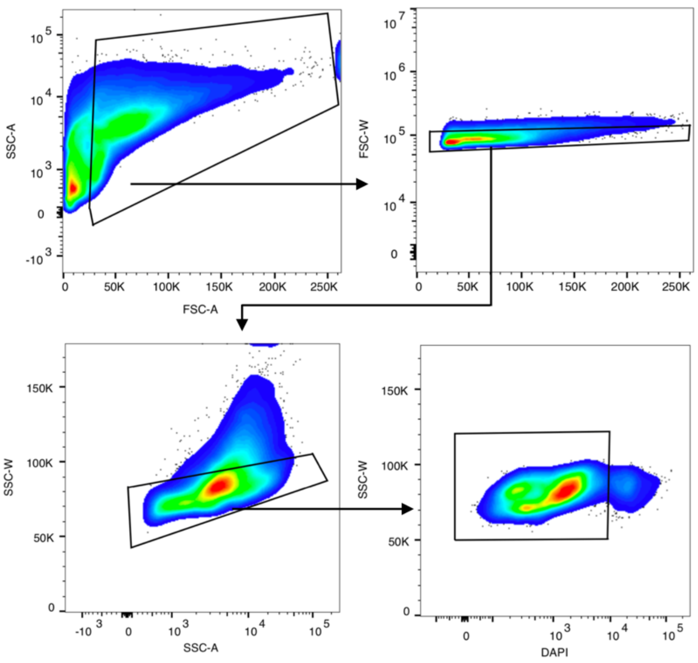

SSC-A
FSC-A
FSC-W
SSC-W
DAPI
0
50K
100K
150K
200K
250K


ADM

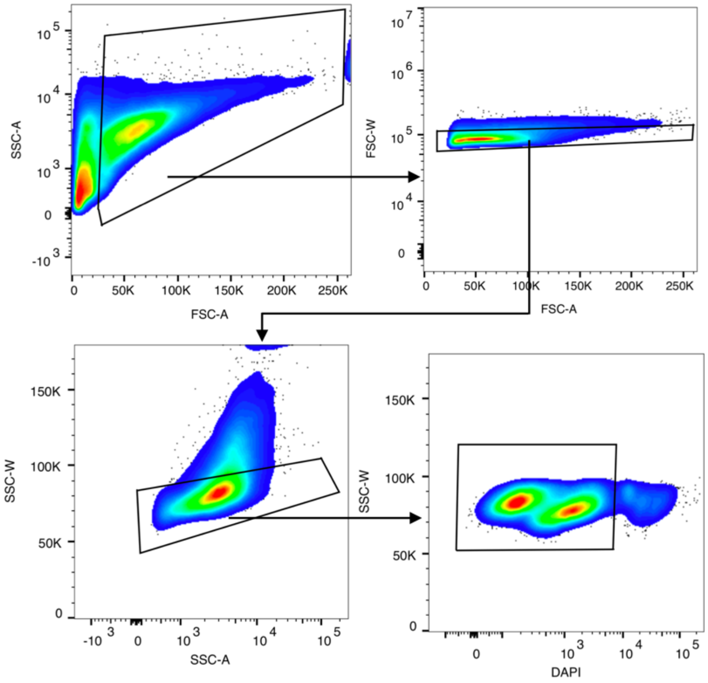

SSC-A
FSC-A
FSC-W
SSC-W
DAPI
0
50K
100K
150K
200K
250K

**Fig. S4.**

Pre-gating strategy for flow cytometry on cells isolated from grafts explanted from parabiosis model (n = 5 pairs per group). hSTSG = human split-thickness skin graft, ADM = acellular dermal matrix graft.

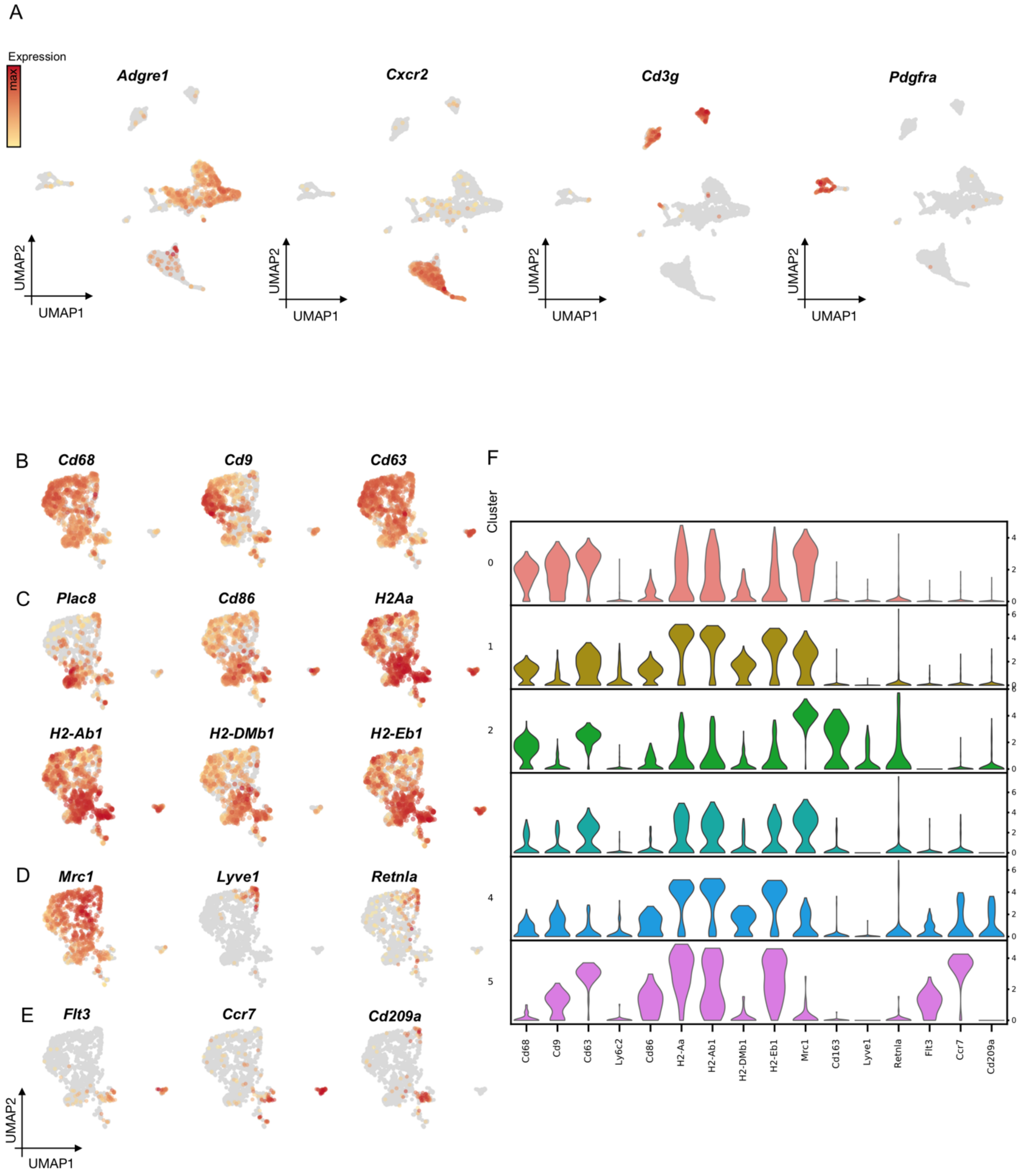

A
Expression
max
Adgre1
Cxcr2
Cd3g
Pdgfra
UMAP2
UMAP1
B
Cd68
Cd9
Cd63
C
Plac8
Cd86
H2Aa
H2-Ab1
H2-DMb1
H2-Eb1
D
Mrc1
Lyve1
Retnla
E
Flt3
Ccr7
Cd209a
F
Cluster
0
1
2
4
5
Cd68
Cd9
Cd63
Ly6c2
Cd86
H2-Aa
H2-Ab1
H2-DMb1
H2-Eb1
Mrc1
Cd163
Lyve1
Retnla
Flt3
Ccr7
Cd209a

**Fig. S5.**

(**A**) Expression of the characteristic cell type marker genes, *Adgre1* (macrophages), *Cxc*r2 (neutrophilic granulocytes)*, Cd3g* (T cells), *Pdgfra* (fibroblasts) projected onto the UMAP embedding. Expression of the characteristic marker genes of myeloid cell subclusters: **(B**) cluster 0, (**C**) cluster 1, (**D**) cluster 2, (**E**) cluster 4 and 5 projected onto the UMAP embedding and (**F**) shown as stacked violin plot.

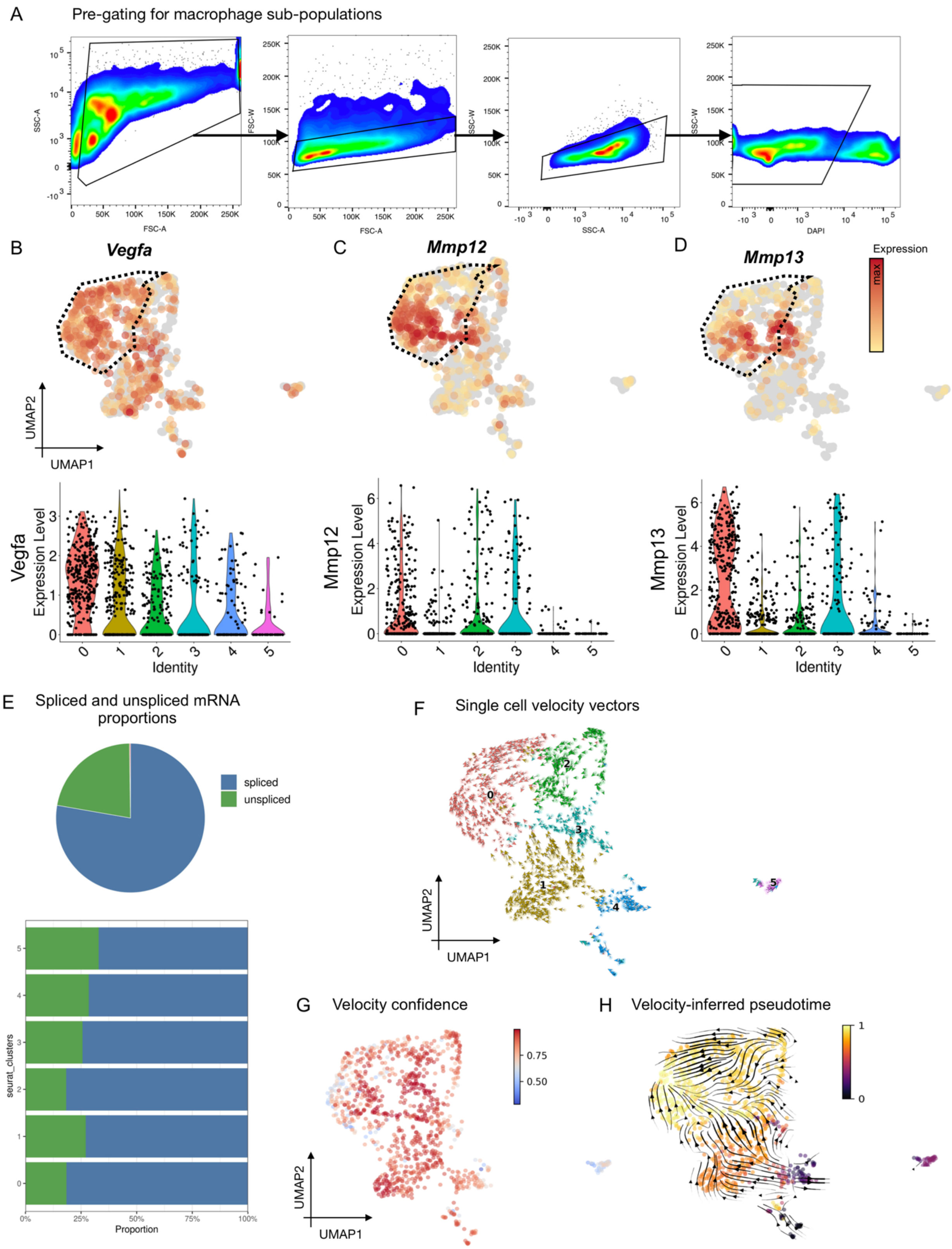

A
Pre-gating for macrophage sub-populations
SSC-A
FSC-A
FSC-W
SSC-W
SSC-A
DAPI
B
Vegfa
C
Mmp12
D
Mmp13
Expression
max
UMAP2
UMAP1
Vegfa
Expression Level
Mmp12
Mmp13
Identity
E
Spliced and unspliced mRNA proportions
spliced
unspliced
seurat_clusters
Proportion
0%
25%
50%
75%
100%
F
Single cell velocity vectors
G
Velocity confidence
0.75
0.50
H
Velocity-inferred pseudotime

**Fig. S6.**

(**A**) Flow cytometry pre-gating strategy for macrophage subpopulations to confirm clusters identified by single-cell RNAseq. (**B**) Expression of *Vegfa* (**C**), *Mmp12*, (**D**) *Mmp13* projected onto UMAP embedding of myeloid cell subset (top row) and shown as violin plot of the 6 clusters (bottom row). (**E**) Ratio of unspliced (green) and spliced (blue) mRNA within the myeloid cell subset (pie chart) and within each myeloid cell cluster, as determined by scVelo. (**F**) Single cell velocity vectors. (**G**) Confidence of RNA velocity analysis. (**H**) Velocity-inferred pseudotime determined by scVelo.

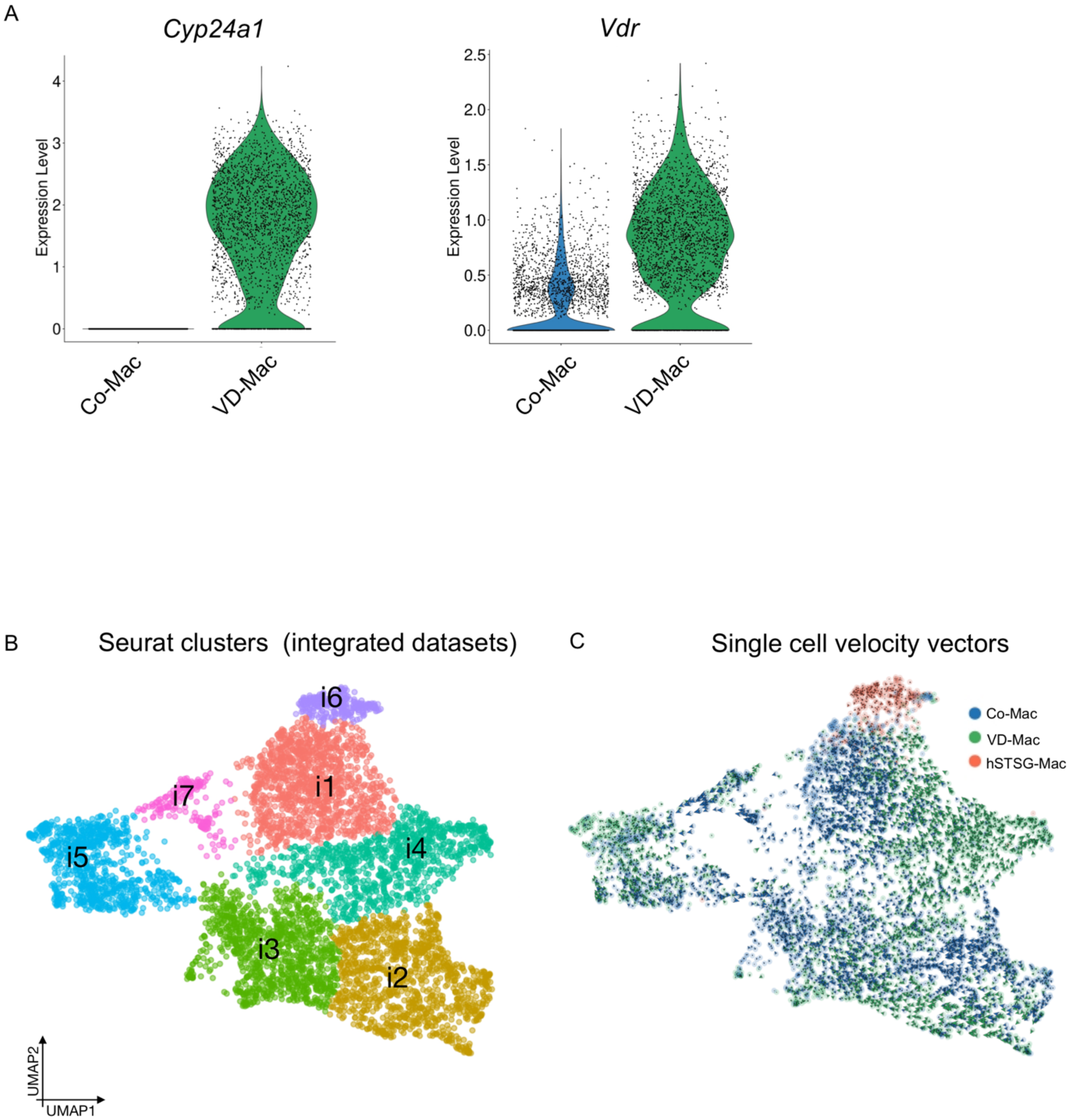

A
Cyp24a1
Vdr
Expression Level
0
1
2
3
4
0.0
0.5
1.0
1.5
2.0
2.5
Co-Mac
VD-Mac
B
Seurat clusters (integrated datasets)
i1
i2
i3
i4
i5
i6
i7
UMAP1
UMAP2
C
Single cell velocity vectors
Co-Mac
VD-Mac
hSTSG-Mac

**Fig. S7.**

(**A**) Expression of *Cyp24a1* (encodes for VD3 24-hydroxylase) and *Vdr* (vitamin D receptor) in control (Co-Mac) and vitamin D3-treated macrophages (VD-Mac). (**B**) 11 distinct cell clusters were identified in the integrated dataset (cultured macrophages integrated with Trem2+ cells (cluster 0) form *in vivo* dataset). (**C**) Single cell RNA velocity vectors generated with scVelo.

A **Brainbow2.1- LyzMcre**

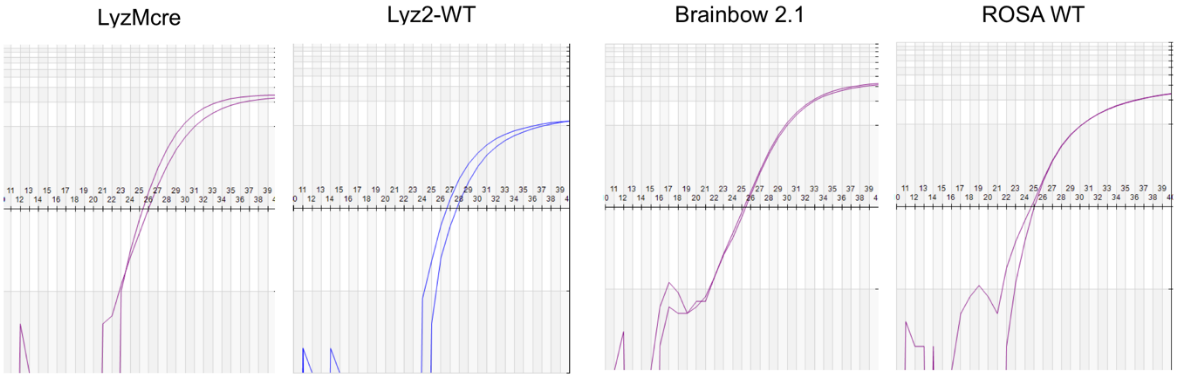


B **Wild-type (C57/BL6)**

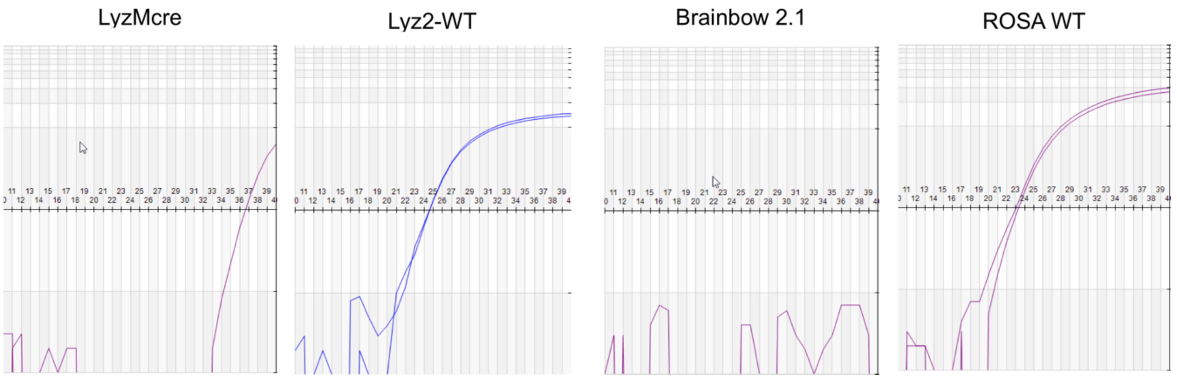


C Unwounded skin, Lyz2M-Cre-Brainbow

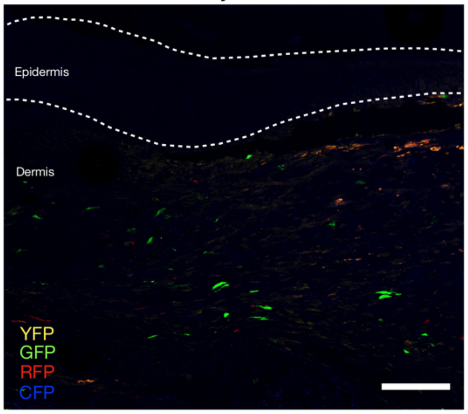

**Fig. S8.**

LyzMcre mice were crossed with R26R-Brainbow 2.1 mice. Exemplary real-time PCR (qPCR) amplification curves confirming the genotype of (**A**) Brainbow 2.1-LyzMcre mouse hemizygous for the LyzMcre and Brainbow 2.1 alleles and (**B**) C57BL6, wild-type (WT) mouse homozygous for the Lyz2 and Rosa WT alleles. X-axis = cycle threshold (CT). (**C**) Unwounded skin of Brainbow 2.1-LyzMcre mouse. Dotted line indicates the location of the epidermis, scale bar = 100 µm.

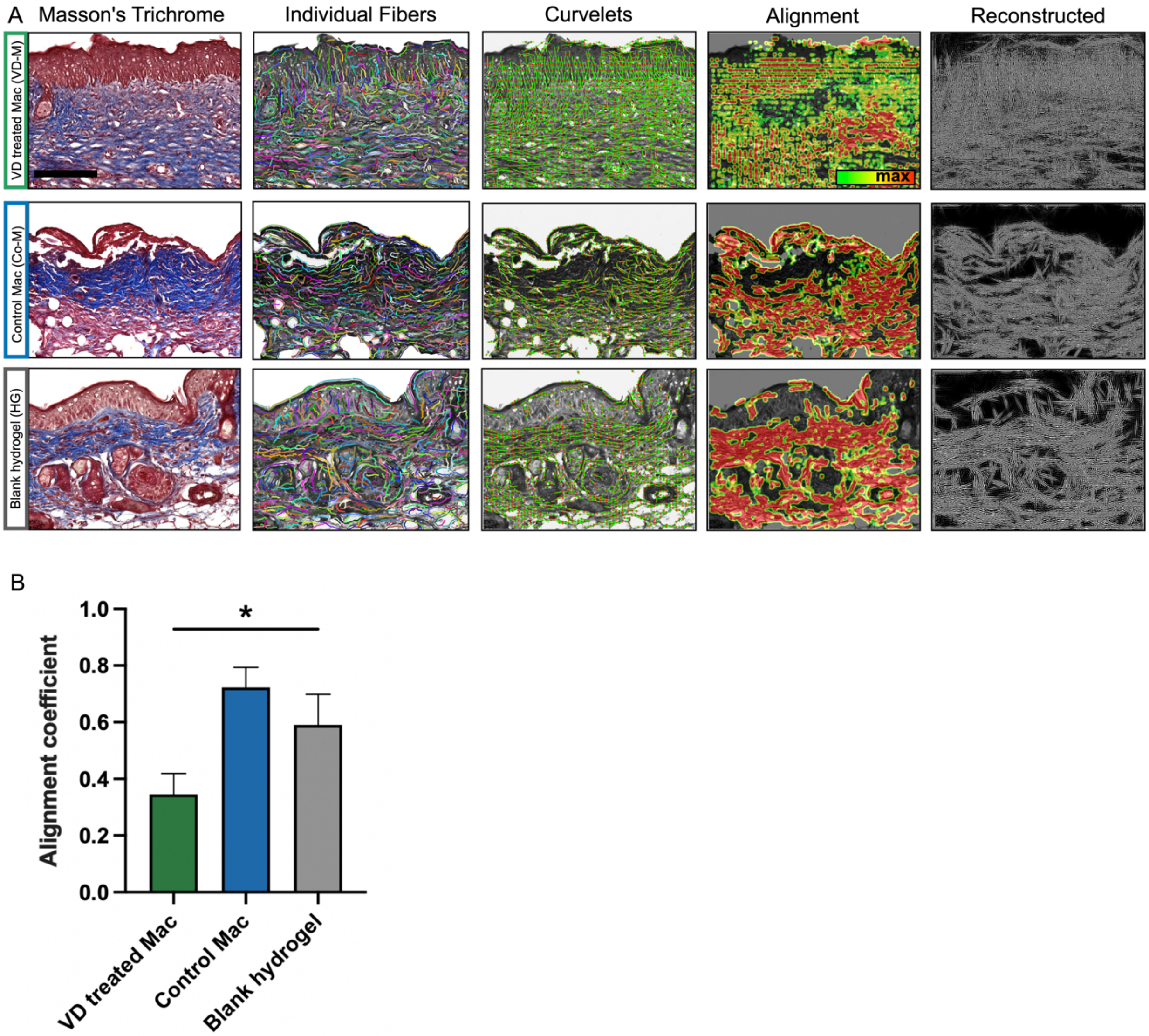
A
Masson's Trichrome
Individual Fibers
Curvelets
Alignment
Reconstructed
VD treated Mac (VD-M)
Control Mac (Co-M)
Blank hydrogel (HG)
max
B
Alignment coefficient
1.0
0.8
0.6
0.4
0.2
0.0
*
VD treated Mac
Control Mac
Blank hydrogel

**Fig. S9** (A) Collagen architecture of wounds treated with vitamin D stimulated macrophages (VD-M, Trem2+), control macrophages (Co-M, bone marrow derived "M1"), and blank hydrogels (HG), from left to right: Masson's trichrome staining; individual fibers identified with CT-FIRE; curvelet transformation as overlay on histologic image (red dots to indicate the center of fiber segments, green lines indicate the fiber orientation at that point); heatmap of alignment with red indicating the regions with the most aligned fiber angles; image reconstructed using CurveAlign; scale bar: 200μm. (B) Comparison of fiber alignment between the groups, One-way ANOVA: *$P < 0.05$. Scale bar = 200μm.

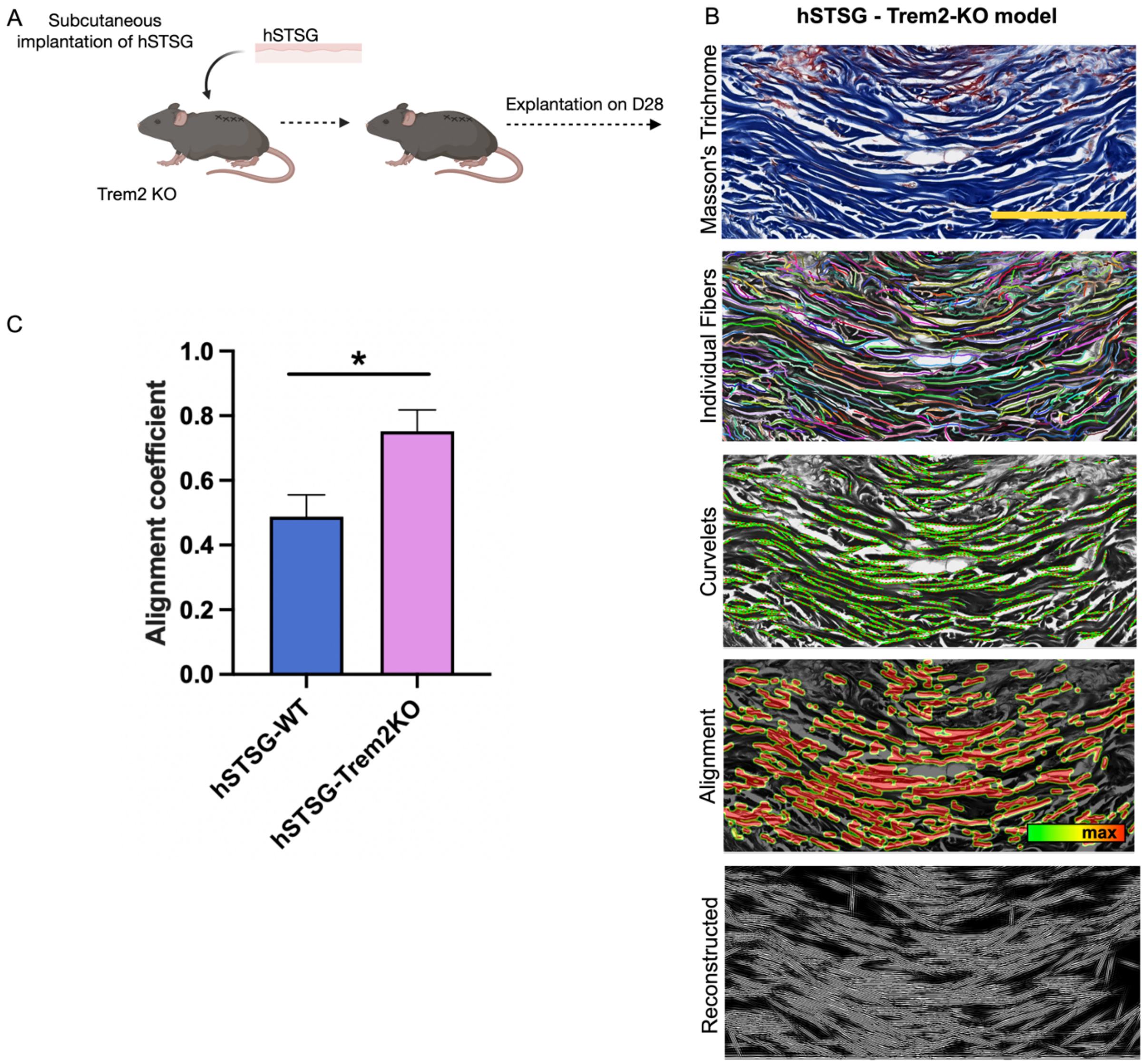
A
Subcutaneous implantation of hSTSG
hSTSG
Trem2 KO
Explantation on D28
B
hSTSG - Trem2-KO model
Masson's Trichrome
Individual Fibers
Curvelets
Alignment
max
Reconstructed
C
Alignment coefficient
1.0
0.8
0.6
0.4
0.2
0.0
*
hSTSG-WT
hSTSG-Trem2KO

**Fig. S10** (A) hSTSG were subcutaneously implanted into Trem2-knockout (KO) mice (n = 5). The grafts were explanted on day 28 for histologic analysis of collagen fiber architecture. (B) Collagen architecture of hSTSG implanted into Trem2-KO mice, from top to bottom: Masson's trichrome staining; individual fibers identified with CT-FIRE; curvelet transformation as overlay on histologic image (red dots indicate the center of fiber segments, green lines indicate the fiber orientation at that point); heatmap of alignment with red indicating the regions with the most aligned fiber angles; image reconstructed using CurveAlign; scale bar: 200µm. (C) Comparison of fiber alignment between grafts implanted into Trem2-KO mice (hSTSG-Trem2KO) vs. grafts implanted into wild-type (WT, C57/BL6) mice. Histology from WT mice is shown in Figure 1D; T-Test: $^*P < 0.05$; hSTSG = human split-thickness skin graft. Scale bar = 200µm.
